# Exploring transcriptomic and genomic differences between susceptible and resistant fetal pigs to maternal PRRSV infection at late gestation

**DOI:** 10.1186/s13567-025-01621-w

**Published:** 2025-11-03

**Authors:** Haesu Ko, J. Alex Pasternak, Paul Stothard, Changxi Li, Graham S. Plastow, John C. S. Harding

**Affiliations:** 1 Western College of Veterinary Medicine, University of Saskatchewan, Saskatoon, SK S7N 5B4 Canada; 2 Department of Agricultural, Food and Nutritional Science, University of Alberta, Edmonton, AB T6G 2H1 Canada; 3 Department of Animal and Food Sciences, University of Kentucky, Lexington, KY 40546-0091 USA; 4 Lacombe Research and Development Centre, Agriculture and Agri-Food Canada, Lacombe, AB T4L 1W1 Canada

**Keywords:** PRRS, gilts, fetus, thymus, RNA-seq, DEG, eQTL

## Abstract

Porcine reproductive and respiratory syndrome (PRRS) severely impacts global swine production. While studies have revealed host genetic factors and molecular mechanisms of fetal response to PRRSV infection, the relationship between fetal genotype, thymic transcription and PRRS susceptibility remains unclear. This study integrates genomic and thymic transcriptomic analyses of fetal pigs from a maternal PRRSV-2 challenge model. Fetuses were categorized by susceptibility to PRRS, following maternal inoculation at gestation day 84–86 into four groups: complete resistance (CR), partial resistance (PR), viable susceptible (VS), and meconium-stained susceptible (MS). CR and PR fetuses exhibited 0 or less than 4 viral loads in serum and thymus, respectively, despite maternal exposure, while VS and MS fetuses had high viral loads with MS showing signs of compromise. Thymic transcriptome analyses revealed a strong interferon response and downregulation of cell cycle and DNA repair pathways in VS and MS fetuses compared to CR and PR fetuses. Severe infection (MS) was further associated with downregulation of genes involved in early thymocyte development, suggesting disrupted T cell development in the thymus. Interaction expression quantitative trait loci (ieQTLs) were identified, where the association between genetic variants and expression levels of differentially expressed genes (DEGs) varied by fetal PRRS susceptibility. Notably, ieQTLs were identified for genes critical for T cell development, including *TMEM98* (involved in Th1 cell differentiation) and *PTCRA* (expressed in early stages of thymocyte development). These findings highlight the complex interplay between fetal genotype, thymic gene expression, and PRRS susceptibility, offering potential genetic markers for breeding programs for replacement gilts.

**Supplementary Information:**

The online version contains supplementary material available at 10.1186/s13567-025-01621-w.

## Introduction

Much progress has been made recently in understanding how fetal pigs with differing susceptibility respond to PRRSV infection through targeted transcriptional analyses [1–6] and high-throughput transcriptomic analysis [7]. These efforts were primarily aimed to elucidate molecular mechanisms of fetal compromise and death in PRRSV-infected gilts by exploring the transcriptional disruptions triggered by PRRSV infection and the corresponding molecular responses. Previous studies highlight the systemic impact of PRRSV, showing altered gene expression in various organs in response to PRRSV infection [1–4, 6]. These studies indicated that fetal pigs with high viral load or meconium-staining respond to PRRSV by upregulating genes for cytokines and chemokines in thymus and spleen [4], and genes related to activation of apoptosis and hypoxia in the thymus, heart, and brain [2], and downregulating cyclin-dependent kinase genes involved in cell cycle transitions across various organs [3, 5]. These findings were observed when comparing fetuses severely affected by the infection to those uninfected in litters from PRRSV-infected gilts. In these studies, uninfected fetuses were considered resistant to PRRSV if virus was not detected in their serum and thymus. However, despite the lack of direct viral detection within uninfected fetuses, subtle transcriptional changes, such as upregulations in leukocyte activator genes or genes associated with pro-inflammatory signaling, were detected in the thymus of uninfected fetuses compared to control fetuses from non-inoculated litters [7]. In the study of Wilkinson et al. [7], 75% of these fetuses had virus detected at the maternal–fetal interface (MFI) which collectively included the endometrium and fetal placental layers. Thus, it may indicate a localized fetal thymic response following PRRSV infection at the MFI in uninfected fetuses. This was also suggested by Malgarin et al. [8] that showed a minimal difference of serum metabolome between fetuses from non-inoculated control gilts and uninfected fetuses from PRRSV challenged gilts.

The thymus plays a crucial role in the maturation of T cells, which are essential for cell-mediated immune protection. During fetal development, T cell progenitors originating from the yolk sac, and later, the fetal liver, migrate to the thymus [9–11]. Here, they undergo processes known as positive and negative selection, ensuring the emergence of functionally competent T cells that can distinguish self from non-self without reacting against the body’s tissues [9–11]. These mature naïve T cells then exit the thymus and populate secondary lymphoid organs such as lymph nodes and spleen to initiate immune response upon antigen exposure [9–11]. Notably, the fetal thymus has been proposed as a principal site for PRRSV replication [12]. This PRRSV replication is also related to an inflammatory and immune-activating environment within the thymus, as evidenced by the upregulation of key transcription factors related to T cell differentiation and function, including T-bet, FOXP3, and EOMES [13], as well as increased expression of antiviral cytokines such as IFN-γ and TNF-α [4, 13]. These molecular changes may reflect an active antiviral and cytotoxic response aimed at controlling PRRSV infection. However, despite this active immune engagement, the overall impact of PRRSV replication in the thymus is immunosuppressive and dysregulatory [14]. It has been demonstrated that PRRSV infection results in a reduction of developing thymocytes and changes in the T-cell receptor (TCR) repertoire in neonatal piglets [15]. Given the pivotal role of the thymus in establishing a competent immune system in fetal pigs [16], understanding how fetal PRRSV infection affects its function is critical.

Host genetic factors related to the fetal response to PRRSV infection have been investigated to better understand the underlying molecular mechanisms of fetal susceptibility or resilience using genome-wide association studies (GWAS) [17, 18], where candidate genomic regions and positional genes have been associated with various fetal PRRS outcomes including viral loads, fetal viability and survival, and thyroid hormone levels. As follow-up, previous research [19, 20] was conducted to validate the effect of variants in a candidate positional gene, *DIO2*, on fetal PRRS outcomes. Nonetheless, there remains an unexplored area in understanding how fetal genotypes affect transcriptional phenotypes in thymus following maternal PRRSV infection, and how this relates to disease susceptibility. The interplay between fetal thymic transcriptional alterations in response to PRRSV infection and genetic predisposition could be key to understanding the mechanisms underlying the severity of PRRSV infection in fetal pigs. As a result, this study was carried out to investigate possible genetic regulatory mechanisms that could explain the susceptibility or resilience of the fetus to PRRSV, by integrating analyses of the fetal thymic transcriptome and the expression quantitative trait loci (eQTLs) using fetal samples from a previously conducted maternal PRRSV-2 challenge trial [21].

We hypothesize that genetic variants associated with variations in gene expression within the thymus across fetuses with different PRRS disease outcomes can help identify candidate genes and biological pathways involved in PRRS susceptibility. We defined fetuses into various groups, considering the neighboring fetal PRRS outcomes due to the observation of clustered fetal compromise and death in inoculated litters [21, 22]. Our study has the following objectives: (1) explore genome-wide expression profiles in four distinct fetal groups representing varying PRRS severities following maternal PRRSV infection and identify genes in the thymus that are differentially expressed between these fetal groups; (2) identify interaction expression quantitative trait loci (ieQTLs) with focus on genetic variants whose association with the expression level of differentially expressed genes (DEGs) varies depending on the fetal group. This analysis helps us identify genetic loci whose effect is potentially modified depending on fetal PRRS susceptibility. Unlike eQTLs which identify genotype-expression associations independent of fetal groups, ieQTLs may reveal context-specific genetic effects critical for understanding differential PRRS disease responses in fetal pigs; and (3) identify candidate genes and biological pathways that might be responsible for variations in fetal susceptibility to PRRS by utilizing the findings from DEGs and ieQTLs.

## Materials and methods

### Animal experiment and sample collection

Fetal samples from a previously conducted pregnant gilt PRRSV-2 challenge trial [21] were used for the present study. The challenge experiment was approved by the University of Saskatchewan’s Animal Research Ethics Board (Protocol #20110102) and was conducted in strict compliance with the guidelines of the Canadian Council of Animal Care. Briefly, purebred Landrace gilts (*N* = 133) sourced from an AcuFast (Spiritwood, SK, Canada) nucleus herd testing clinically and serologically negative for PRRSV, *Mycoplasma hyopneumoniae*, and *Actinobacillus pleuropneumoniae*, were used in a total of 12 experimental batches (3–15 gilts per batch). In each batch, estrus synchronized gilts were bred using a single-sire breeding strategy with semen sourced from 24 purebred Yorkshire boars. Pregnant gilts were randomly assigned to either the PRRSV-2-inoculated (INOC) or control (CTRL) group. At gestation day 84–86, INOC gilts (*N* = 114) were challenged with 1 × 10^5^ TCID_50_ PRRSV-2 isolate (NVSL 97-7895) in 4 mL minimum essential medium (MEM) delivered 50/50 via intramuscular and intranasal routes, while CTRL gilts were similarly inoculated with MEM. Gilts and fetuses were humanely euthanized on day 21 post-inoculation (D21). Fetal pigs were removed from the uterus and dissected to obtain fetal tissues and assess fetal preservation. Collected fetal tissues included blood from the axillary artery, thymus, spleen, lung, heart, liver, and brain. Blood was allowed to clot, then centrifuged to obtain the serum. All the tissue and serum samples were stored at −80 °C.

### Experimental group selection

**Figure 1:**
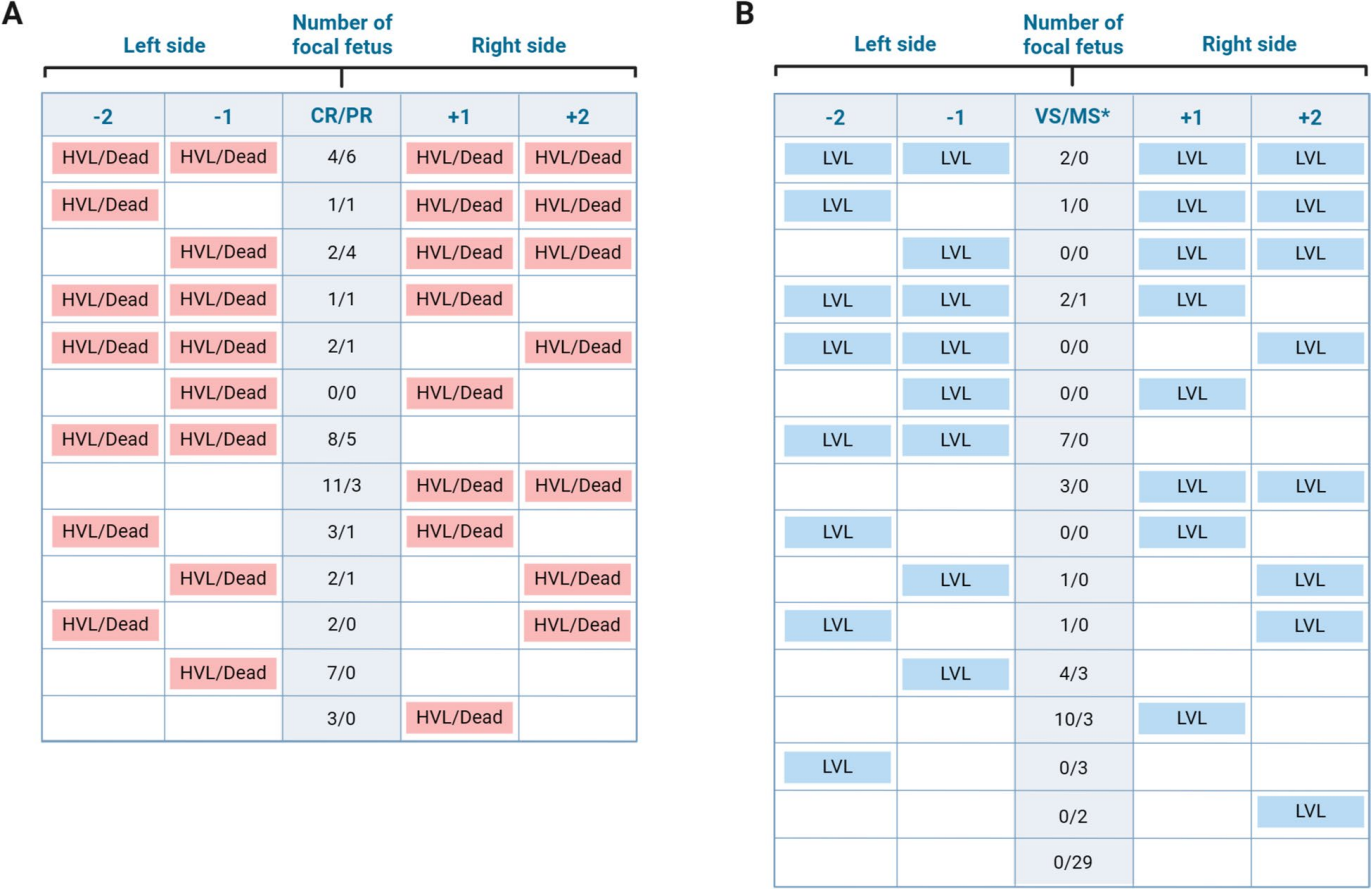
**Fetal group assignment considering in-utero preservation, viral loads, and adjacent fetus status.** Each cell represents a fetus, with the center cells denoting the assigned group: Complete Resistance (CR), Partial Resistance (PR), Viable Susceptible (VS), or Meconium-stained Susceptible (MS). The x-axis represents the position of the fetus relative to the focal fetus: left fetus 2 (−2), left fetus 1 (−1), focal fetus (CR/PR or VS/MS), right fetus 1 (+ 1), right fetus 2 (+ 2). (A) For resistant fetuses (CR/PR), at least one adjacent fetus is dead or has a high viral load (HVL). (B) VS fetuses have at least one adjacent fetus with a low viral load (LVL) but viable. *83% (34/41) of fetuses in the MS group did not meet the selection criteria for neighboring fetal conditions. Edited with BioRender.

Fetuses were classified into the following groups based on in utero fetal preservation observed during necropsy: viable (VIA), meconium-stained (MEC), decomposed (DEC), autolyzed (AUT), and mummified (MUM) [21]. A subset of the live (VIA and MEC) fetuses was selected for the fetal group signifying varying degrees of PRRS susceptibility from the entire fetal population (*N* = 1422 including dead fetuses) from 111 INOC gilts (fetuses from non-inoculated gilts were not used for this study). The live fetuses were further classified into four different resistance groups: (1) complete resistance (CR), (2) partial resistance (PR), (3) susceptible viable (VS), and (4) meconium-stained susceptible (MS) based on in utero fetal preservation, viral loads in maternal–fetal interface (MFI) including endometrium and fetal placenta, and fetal serum (SER) and thymus (THY), together with fetal preservation scores and viral loads of fetuses adjacent to the focal fetus. PRRSV RNA concentration in MFI, SER, and THY were quantified using a probe-based quantitative real-time PCR assay targeting ORF7, as detailed in [21]. Then, PRRSV RNA copies per µL SER or mg THY were used to categorize low viral load (LVL; < 4 log_10_ copies) or high viral load (HVL; ≥ 4 log_10_ copies). For each fetus, its neighbors were defined as the closest fetus and the second-closest fetus adjacent to it, whether on its left or right side. Fetuses were assigned as CR if they were VIA, had no detectable virus in SER and THY even though the virus was detected in their respective MFI, and at least one neighboring fetus that was dead (DEC or AUT) or had HVL in SER or THY (greater than or equal to 4 log_10_ PRRSV RNA copies). Fetuses having no detectable virus in THY and LVL in SER (less than 1 log_10_ PRRSV RNA copies) were considered as CR since we assumed that virus detection in thymus was the minimum condition required for a differential transcriptional response in THY. PR fetuses were VIA, had LVL in both SER and THY, and at least one neighboring fetus that was dead (DEC or AUT) or had HVL in SER or THY. If fetuses were VIA, but had HVL in both SER and THY, and at least one neighboring VIA fetus that was LVL, they were assigned to VS. The same selection criteria applied to MS group, except their fetal preservation was MEC. A schematic representation of the selection criteria in relation to neighboring fetal conditions is shown in Figure 1.

### Preprocessing genomic DNA genotypes

Genotypes were obtained for the specific fetuses selected for this study from a dataset analyzed in a previous GWAS by Yang et al. [17]. Genotypes of 61 565 SNPs (60 K SNPs) from the Illumina PorcineSNP60 BeadChip v2 were analyzed for 928 fetuses with thymic DNA. Quality control (QC) was conducted using plink (v1.9) [23, 24], applying filtering thresholds used in previous porcine eQTL studies [25–30], including GWAS for the pregnant gilt PRRSV-2 challenge model [17, 18]. Autosomal SNPs were retained if they had a genotyping rate ≥ 90%, *P* value ≥ 1 × 10^–10^ for Hardy–Weinberg exact test, and minor allele frequency ≥ 5%. Additionally, 11 duplicate SNP sites across autosomes were removed. Fetuses were excluded if they had inconsistency between sex determined using X and Y chromosomal SNPs, and sex determined during necropsy (*N* = 3), or a missing genotyping rate > 10% (*N* = 1). After QC, the dataset comprised 43 113 SNPs and 141 fetuses.

### RNA extraction, sequencing, and preprocessing RNA-seq reads

RNA sequencing was conducted to measure the abundance of all expressed genes in fetal thymus as previously described [7]. Briefly, total RNA was extracted from fetal thymic tissues frozen with liquid nitrogen using the AllPrep RNA/DNA isolation Mini kit (Qiagen, Hilden, Germany), following the manufacturer’s protocol. RNA quantity and quality were assessed using a Nanodrop ND 2000 (Thermo Fisher Scientific, Waltham, USA) and a 2200 Tapestation (Agilent Technologies, Santa Clara, USA), respectively. One µg of total RNA was used to generate cDNA libraries using the TruSeq RNA sample preparation kit v2 (Illumina, San Diego, USA), and the resulting libraries were sequenced using the TruSeq SBS kit v3-HS (Illumina) on a HiSeq 2000 (Illumina) to a median depth of 48.5 million paired-end reads per sample, at the Genome Quebec Innovation Centre (McGill University, Montreal, Canada). Paired-end 100 bp reads were produced across all fetal samples. Raw RNA-seq read quality was assessed using FastQC (v.0.11.9). Quality and adapter trimming of raw sequencing reads were performed with Trim Galore (v.0.6.7) using a Phred quality threshold of 20 (–q 20), adapter removal with a minimum overlap of 7 bp (–stringency 7), and discarding reads shorter than 20 bp (–length 20). Paired-end reads were processed in paired mode (–paired), with FastQC applied to both raw and trimmed reads for quality control (–fastqc). The sequencing quality of all fetal samples before and after trimming was compared using MultiQC (v1.6) [31]. No fetal samples were removed after read QC, and all trimmed RNA-seq reads were used for all downstream analyses.

### Variant calling from RNA-seq reads

RNA-seq variant analyses were performed using the nf-core/rnavar pipeline [32] to map trimmed reads to the porcine reference genome (Sscrofa11.1) and preprocess mapped reads for the variant calling step. Then, the Genome Analysis Toolkit (GATK) joint genotyping workflow [32, 33] was applied to detect RNA-seq variants using nf-core/sarek pipeline [32]. Distributions of variant quality metrics were plotted for each variant type (SNP, INDEL, MIXED), then filtering of RNA-seq variants was conducted using gatk (v4.2.4.0) based on the GATK recommendations for each variant type, separately as follows: (1) SNP: QD < 2.0, FS > 60.0, SOR > 3.0, ReadPosRankSum < −8.0; (2) INDEL and MIXED: QD < 2.0, FS > 200.0, ReadPosRankSum < −20.0. The numbers of SNP, INDEL, and MIXED variants that met all filtering thresholds (i.e., PASS-flagged variants in the FILTER field from the variant dataset) were 980 456 (93.737%), 693 121 (94.617%), and 115 093 (86.704%), respectively. Then, biallelic SNP and INDEL with PASS-flags were subsequently filtered out using bcftools (v1.18) and plink (v1.9) according to the following criteria: per-sample depth, DP < 3 or genotype quality, GQ < 20, located on sex chromosomes, the fraction of missing genotypes over 10%, and minor allele frequency below 5%. There were no biallelic sites for the MIXED variant type, so it was not selected for downstream analyses. For sample level filtering, samples were removed due to possible sample mix-ups (*N* = 3) based on inconsistency between assigned sex and sex determined by SNPs, which were identified from the porcine 60 K SNP chip data, a missing genotype rate over 10% across variants (*N* = 3), or lack of genomic DNA genotypes (*N* = 1). After QC, the RNA-seq variant dataset comprised 74 481 SNPs and 12 546 indels for 138 fetal pigs.

### Transcriptome analysis

To profile gene-level expression in fetal thymus, Rsubread (v2.10.5) [34] was used to map trimmed reads to the porcine reference genome (Sscrofa11.1, Ensembl release-107), achieving an average mapping rate of 98%, and to count the number of mapped reads that overlap with any feature of annotated genes (Ensembl release-107) in R (v4.2.1). Gene annotation data was also provided for the align function to increase accuracy in mapping junction reads. Multi-mapping reads and multi-overlapping reads were not counted. Overall, the gene transcription abundance was summarized as the number of reads aligning to the gene (*N* = 35 670) in each fetal sample (*N* = 141). Then, common workflows of transcriptomic data analysis were performed using R packages described in the framework of “tidybulk” (v1.14.2) [35] in R (v4.3.2). Read counts were filtered based on a minimum count threshold of 10 in at least 70% of the samples within each fetal group and a minimum total count of 15 across all samples. Genes that met these criteria were considered expressed. These genes were then normalized using the trimmed mean of M-values (TMM) algorithm [36] to compensate for differences in sequencing depth between samples.

To understand variability in the thymic transcriptome in fetuses with differing PRRS outcomes, principal component analysis (PCA) was conducted using read counts normalized by the TMM method for the 500 most variable genes across samples, and a variance component model based on ANOVA was fitted to understand the contributions of experimental factors (fetal group, fetal sex, experimental batch, sire, and dam) to the overall variability in the top three principal components (PC). Additionally, gene set co-regulation analysis (GESECA) was performed to determine whether a given set of genes annotated with biological pathways was significantly associated with the variance in thymic gene expression across fetuses, using fgsea (v1.28.0) [37] in R (v4.3.2). This analysis identifies co-regulated expression patterns of genes within predefined gene sets (representing biological pathways or processes), where genes with significantly correlated expressions within each gene set explain the variance in the fetal thymic transcriptome. In this analysis, genes with highly correlated expressions within each gene set led to a high gene set score, while uncorrelated gene expressions within the set resulted in a low gene set score. Log_2_ CPM values were normalized by the TMM method, adjusted for fetal sex and batch to control for unwanted variations in gene expression levels using the ComBat-seq method [38], and then centered across fetuses for each expressed gene in fetal thymus before calculating gene set scores. Gene sets were analyzed using the hallmark gene sets (H collection) and curated gene sets (C2 collection) for humans from the Molecular Signatures Database (MSigDB) [39–41]. A total of 3955 genes were associated with at least one hallmark gene set, and 14 022 genes were linked to at least one curated gene set from the MSigDB collection. *P* values for gene set scores were estimated by adaptive multilevel splitting Monte Carlo approach [37]. The significance of the gene set scores was determined at Benjamini–Hochberg (BH)-adjusted *P* value < 0.05.

### Analysis of differential gene expression

All analyses were executed in R (v4.3.2). Analysis of differential gene expression was conducted using the edgeR (v4.0.14) quasi-likelihood pipeline [42, 43]. Based on the PCA and variance component analysis conducted in the transcriptome analysis, fetal sex and experimental batch were included in the models testing for differential gene expression between fetal groups, to eliminate their systematic influence on thymic gene expression. Four fetal groups (CR, PR, VS, MS) were compared to identify differentially expressed genes (DEGs) through six a priori pairwise comparisons: PR versus CR, VS versus CR, MS versus CR, VS versus PR, MS versus PR, MS versus VS. DEGs were defined at a BH-adjusted *P* value < 0.05 with the absolute value of log_2_ fold change (FC) greater than or equal to 1. A FC of 2 to 2.4 was estimated to be reliably detected at a power of 0.8, depending on the sample size of each fetal group, based on RNA-seq power analysis using RNASeqPower (v1.42.0) [44]. To capture a broader range of significant DEGs, a FC of 2 was set to define DEGs. Gene set enrichment analysis (GSEA) was conducted for all thymic expressed genes tested within each DEG contrast using fgsea (v1.28.0) [37]. Genes were preranked based on their differential expression using log_2_ FC and nominal P values in each contrast. The curated gene sets (BioCarta, KEGG, PID, Reactome, and WikiPathways, *N* = 2289) for humans from the MSigDB [39–41] were tested for enrichment in these preranked gene lists. Significant enrichment of pathways was determined at BH-adjusted *P* value < 0.01.

### Examination of the expression levels of thymopoiesis-associated genes across fetal groups

All analyses were conducted in R (v4.3.2). Using a published single-cell RNA sequencing dataset of the thymus merged from two mixed-breed pigs aged 22 weeks [45], thymocyte markers and gene sets corresponding to successive stages of thymopoiesis were selected. These gene sets, consisting of 16 modules based on pseudo-temporal analysis, reflect distinct developmental stages of specific thymocyte subsets, except modules 14–16, as follows: developing thymocytes (double negative thymocytes [module 1] and positive thymocytes either at cell cycling [module 2–5] or quiescent [module 6] stage); post-committed thymocytes (conventional T cells, CD4+/CD8+ T cells [module 7]; unconventional CD8+ T cells, interferon-stimulated gene (ISG)-CD8 T cells [module 8], CD8αα cells [module 10], cytotoxic CD8 T cells [module 11]; T regulatory cells [module 9], and CD2+ [module 2,7,12] and CD2-γδ T cells [module 7,12,13]). Modules 14–16 were excluded as they do not correspond to specific thymocyte subsets [45]. Subsequently, GESECA was conducted to test for association of these gene sets with variance in the thymic gene expressions using fgsea (v1.28.0). Furthermore, DEGs identified in our analysis were individually compared with the thymocyte marker genes and genes in the module gene sets, and these comparisons were visualized with box and dot plots using the ggplot2 [46]. GSEA was also performed to test for enrichment of thymocyte module gene sets (*N* = 16) from Gu et al.’s study [45] using fgsea (v1.28.0). Significant enrichment of the gene sets was determined at BH-adjusted *P* value < 0.1.

### eQTL mapping analysis for DEGs

Gene expression levels for DEGs from 46 CR, 23 PR, 31 VS, and 41 MS (38 MS for RNA-seq variants) fetal pigs were tested for nominal genotype-by-fetal group associations in a univariate linear mixed model using GEMMA (v0.98.5) [47]. Read counts, normalized by the TMM method, were adjusted for fetal sex and experimental batch using ComBat-seq method [38] to avoid any possible confounding effects. The adjusted counts were then converted to log_2_ CPM values for use as gene expression levels. The log_2_ CPM values were visually inspected to assess their normality and outliers. Any missing genotypes from the 60 K SNPs and RNA-seq variants after genotype QC were imputed with phased genotypes using SHAPEIT2 [48], which was required for eQTL mapping analysis. The following linear mixed effects model was employed, incorporating an interaction term to investigate potential genotype-by-fetal group interactions:

$${\text{y}}_{{{\text{ijk}}}} =\upmu + {\text{V}}_{{\text{j}}} + {\text{G}}_{{\text{k}}} + {\text{V}}_{{\text{j}}} {\text{G}}_{{\text{k}}} + {\text{a}}_{{{\text{ijk}}}} +\upvarepsilon _{{{\text{ijk}}}} ,$$

where y_ijk_ represents the gene expression level of a DEG of the individual i (i.e., adjusted counts converted to log_2_ CPM values) with genotype for the jth variant (V_j_) in fetal group k (G_k_), and μ is the intercept. V_j_ is the fixed effect of the jth variant genotype (3 levels; 0,1,2 coded), derived from 60 K SNP data or RNA-seq data. G_k_ is the fixed effect of the kth fetal group (4 levels; CR, PR, VS, MS). V_j_G_k_ is the interaction effect between genotype of the jth variant (V_j_) and the kth fetal group (G_k_). a_ijk_ is the ijkth animal random additive genetic effects. ε_ijk_ is the residual errors associated with the ijkth fetus. a_ijk_ ~ MVN_n_(0, λτ^−1^ K) indicates that the random effects follow a multivariate normal distribution with a mean of 0 and a covariance matrix defined by λτ^−1^ K, where λ is the ratio between the variance of random additive genetic effects and the variance of the residual errors, τ^−1^ is the variance of the residual errors, and K is the animal relatedness matrix, which was estimated using GAPIT (version 3) in R (v4.3.2) based on the 43 113 SNPs from the 60 K SNP data or 74 481 SNPs and 12 546 indels from RNA-seq data as proposed by VanRaden [49] to account for the genetic relatedness or background genetic effects between fetal pigs within litters and sires while a single DNA variant is tested for associations with the gene expression level. ε_ijk_ ~ MVN_n_(0, τ^−1^I_n_) indicates the errors also follow a multivariate normal distribution, with an identity matrix (I_n_). The BH procedure was applied to adjust for multiple comparisons, and the likelihood ratio test was used to test for the statistical significance of the interaction effect. A BH-adjusted *P* value threshold of < 0.05 was used to identify significant interaction eQTLs (ieQTLs) associations. Significant ieQTLs were annotated for predicted functional impact on genes, transcripts, and protein sequences using the Variant Effect Predictor [50], based on Ensembl release-110. This information was then used to identify positional candidate genes for significant ieQTLs. ieQTLs were selected within a 1 megabase (Mb) region flanking each tested DEG to analyze interaction cis-acting SNPs or indels as described by [51, 52], while ieQTLs outside this region were excluded from downstream analyses. The 1 Mb flanking region was selected as a relaxed threshold to support the identification of potential cis-regulatory variants, rather than strictly following pig-specific linkage disequilibrium decay distances (~100–500 kb, per Badke et al. [53]). Genes with at least one significant ieQTL were designated as ieGenes. ieGenes were functionally grouped using the curated gene sets for humans from the MSigDB [39–41].

### Post-GWAS marker analysis

Post-GWAS analysis was conducted on selected ieGenes in R (v4.3.2) to investigate how the effect of selected ieQTL variants on DEG expression varied across different fetal groups. Conditional residuals were obtained from the null eQTL model (y_ijk_ − μ − a_ijk_), to adjust for the animal random genetic effects, using GENESIS (v2.32.0). Linear regression models were constructed with conditional residuals as the response variable. Predictor variables included the genotype at each selected ieQTL, fetal group, and an interaction term between genotype and fetal group. Pairwise comparisons of estimated marginal means (EMMs) across different genotypes within each fetal group were performed using emmeans (v1.10.0). The BH method was applied to control false discovery rate, adjusting *P* values across all genotype and fetal group contrasts. A BH-adjusted *P* value < 0.05 was considered statistically significant, while 0.05 ≤ *P* < 0.10 indicated a suggestive trend. Additionally, positional candidate genes for ieQTLs were compared with candidate genes at GWAS QTLs that were significantly associated with fetal PRRS disease phenotypes including viral loads in thymus [17], fetal viability and survival [17], and thyroid hormone levels [18]. Lastly, linkage disequilibrium between selected ieQTLs was estimated using snpStats (v1.52.0).

## Results

### Description of fetal groups

Of 145 fetuses that met the fetal group selection criteria, 4 failed genomic DNA QC, and 7 failed RNA-seq QC. The median viral loads in MFI, SER, THY across fetal groups showed the trend expected for these criteria (Table 1). CR had the lowest viral loads, with a gradual increase through PR and VS, and the highest in MS. Fetal weight ranged from 237.2 to 1675.9 g across fetal groups, with the numerically lowest median weight observed in the VS group (913.0 g) and the highest in the MS group (1031.8 g). Median crown rump length was very similar across all groups, ranging from 20.8 to 33.5 cm. MS fetuses had numerically the lowest median brain to liver ratio, which is consistent with results showing fetuses with lower brain to liver ratio from the mean are more likely to be classified in the high viral load group [54]. A few fetuses did not strictly meet the selection criteria for each fetal group. For example, two PR fetuses had zero VL in THY but had virus detected in SER (1.7, 2.1 VL, respectively), 1 PR fetus had zero VL in MFI, and 1 VS fetus had 3.9 VL in SER but 5.3 VL in THY. Most fetuses in the MS group did not have at least one adjacent VIA and LVL fetus since most of the neighboring fetuses to the MEC fetus were inherently MEC, VIA with HVL, or dead.

**Table 1:** **Median (range) for variables used in fetal group selection along with fetal morphometric features**

Fetal group	N	Fetal preservation	Viral load in maternal–fetal interface	Viral load in serum	Viral load in thymus	Fetal weight (g)	Crown rump length (cm)	Brain to liver ratio
CR	46	VIA^*^	3.8 (0.6–5.8)	0.0 (0.0–0.9)	0.0 (0.0–0.0)	1021.8 (237.2–1645.6)	29.2 (20.9–33.5)	1.1 (0.7–2.2)
PR	23	VIA	3.3 (0.0–5.9)	1.1 (0.1–3.7)	1.8 (0.0–3.7)	962.2 (547.0–1309.7)	28.1 (23.1–31.3)	1.2 (0.7–2.0)
VS	31	VIA	5.0 (1.3–7.3)	6.1 (4.6–7.5)	6.8 (3.9–8.0)	913.0 (390.4–1675.9)	28.4 (21.4–33.0)	1.3 (0.6–3.8)
MS	41	MEC^**^	6.1 (4.2–7.2)	6.7 (5.0–7.6)	7.2 (5.1–8.2)	1031.8 (425.0–1492.9)	28.2 (20.8–32.1)	0.7 (0.5–1.6)

^*^Viable.

^**^Meconium-stained.

### Thymic gene expression profiles across fetuses

**Figure 2:**
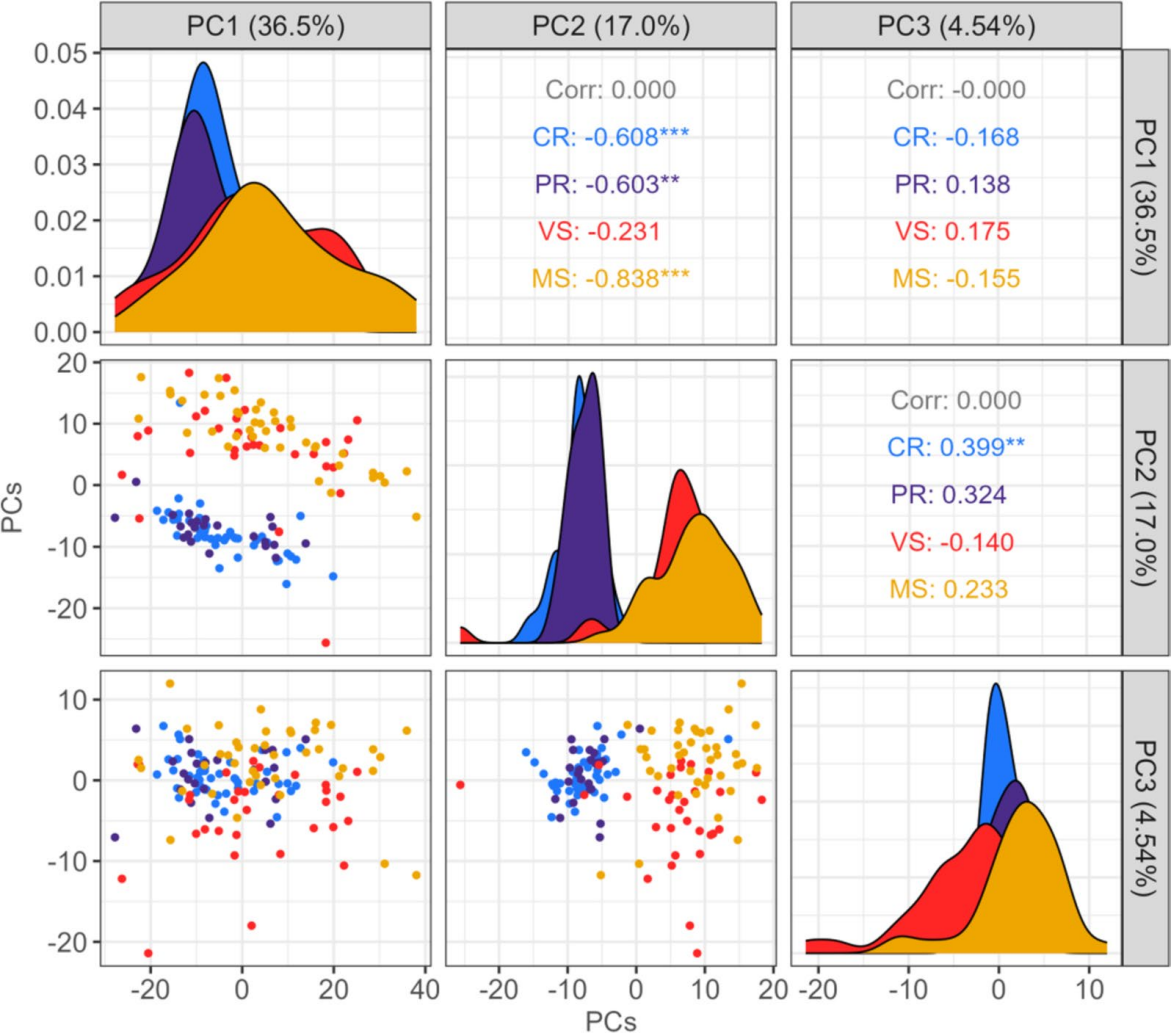
**Pair plot of the first three reduced dimensions for fetal thymic transcriptome using PCA.** The first three principal components (PCs) were estimated using read counts for the 500 most variable genes. Read counts were normalized and adjusted for fetal sex and experimental batch. Correlation coefficients between pairs of PCs were displayed in the upper triangle; scatter plots of each pair of PCs were shown in the lower triangle; the distribution of each PC was displayed within each fetal group in the diagonal. Colors denote each fetal group; light blue, Complete Resistance (CR); purple, Partial Resistance (PR); red, Viable Susceptible (VS); gold, Meconium-stained Susceptible (MS).

Across all fetuses, 18 100 genes were expressed in the thymus. We employed PCA, variance component analysis, and GESECA to understand overall variability in thymic gene expressions across all fetuses. Initial PCA of the normalized read counts for the 500 most variable genes revealed that the first three PCs explained 60.7% of the variance. Importantly, PCA demonstrated a clear separation between resistant (CR/PR) and susceptible (VS/MS) fetuses along PC2 (Figure 2). This robust separation implied distinct transcriptional responses associated with differences between resistant and susceptible fetal groups.

To quantify the possible drivers of this variation, we performed variance component analysis. Fetal group was significantly associated with each PC (PC1: *P* = 0.006, PC2: *P* < 0.001, PC3: *P* < 0.001), with a substantial portion of PC2 variance (79.3%) explained by fetal group. This further supported the impact of fetal group on thymic gene expression differences. Notably, PC3 showed significant associations with fetal sex, experimental batch, and sire, where each variable explained 32.3%, 11%, and 13.9% of the variance in PC3, respectively. After adjustments for fetal sex and batch, the distinct separation was maintained between resistant and susceptible groups on PC2, and it revealed some separation within the susceptible fetuses (VS, MS) along PC3 (Figure 2).

We conducted GESECA to identify the biological pathways whose genes show strong positive co-regulation in the fetal thymus, thereby revealing which pathways most influence the overall transcriptomic variability in our dataset. Results from the GESECA using the hallmark gene sets revealed 29 key biological pathways associated with 3063 genes significantly driving variability in the fetal thymic transcriptome. The top 20 most significant pathways included those involved in myogenesis, interferon responses, cell cycle regulation, and inflammation. Several pathways, such as interferon gamma and alpha response (Number of genes (*N*) = 183 and *N* = 88, respectively), inflammatory response (*N* = 186), and TNFα signaling via NFκB (*N* = 189), play critical roles in immune system function and response (Additional file 1). This analysis also identified genes involved in apoptosis (*N* = 156) and hypoxia (*N* = 187) as significant contributors to the variation in fetal thymic gene expression. Curated gene set analysis from the GESECA further highlighted the importance of immunological processes in driving fetal thymic gene expression variance. This included significant contributions from genes involved in neutrophil degranulation (*N* = 433), interferon signaling (*N* = 164), cytokine-cytokine receptor interaction (*N* = 193), and cell cycle checkpoints (*N* = 252) (Additional file 2). Complete gene lists for the top 20 most significant pathways among the hallmark gene sets and the curated gene sets are provided in Additional file 3. Patterns of Changes in gene expression within the significant gene sets across fetal groups also supported these findings. For instance, interferon responses were the main immune process differentiating resistant versus susceptible fetuses. The co-regulation profiles of genes within the top 3 hallmark pathways are summarized in Additional file 4. The myogenesis-related gene set displayed greater variability across individual samples, whereas the interferon response gene sets showed more uniform expression patterns within each fetal group, resulting in more pronounced group-level differences.

Overall, these analyses identified similarity in thymic gene expression profiles within the resistant groups (CR, PR) or the susceptible groups (VS, MS), and differentiation between the resistant and susceptible groups, based on both the PCA clustering or the broader gene expression patterns in relation to key biological pathways captured by the GESECA. GESECA validated key pathways that had been mainly targeted in previous gene expression studies [2–5], further supporting their involvement in fetal response to PRRSV.

### Differentially expressed genes (DEGs) between fetal groups

**Figure 3:**
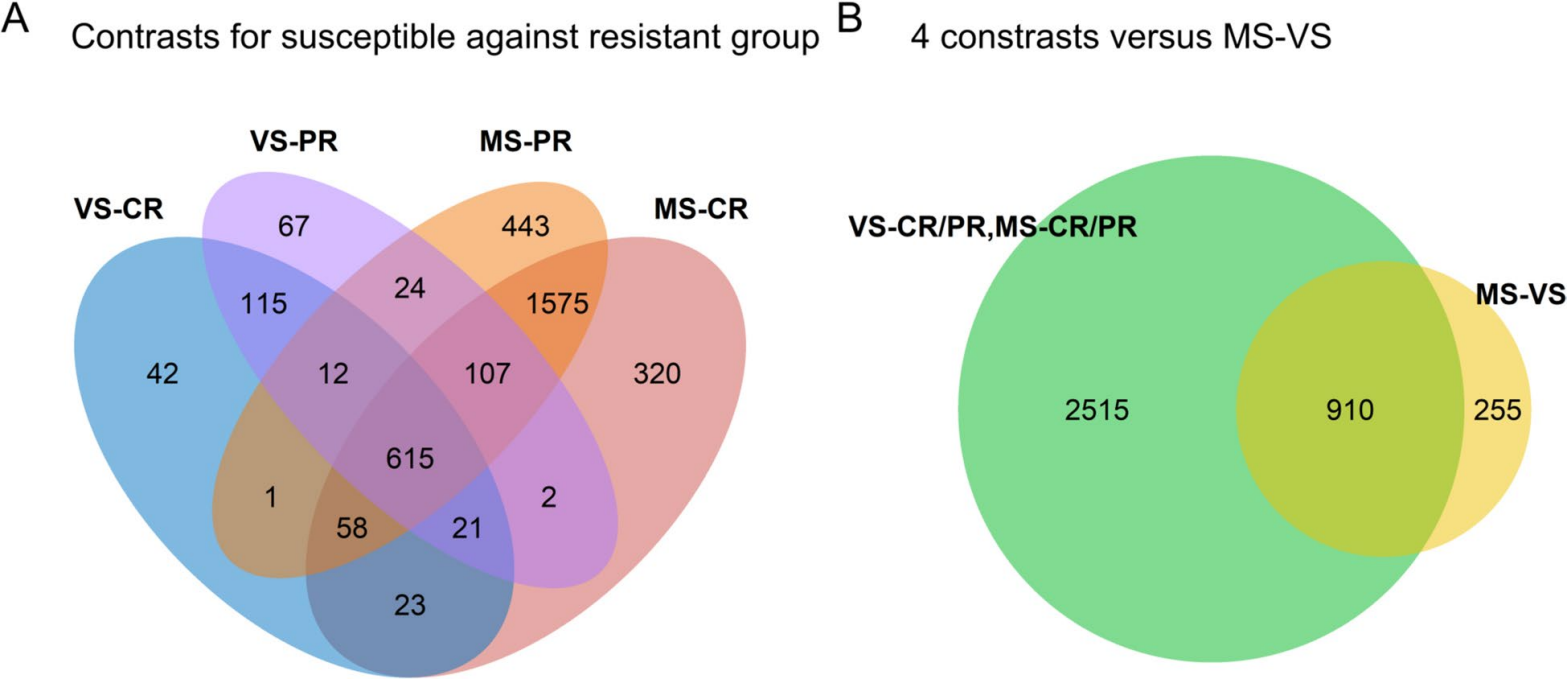
**Venn diagrams showing unique or shared DEGs among the 5 contrasts.** **A** Susceptible fetal groups [Viable Susceptible (VS), Meconium-stained Susceptible (MS)] compared against resistant fetal groups [Complete Resistance (CR) or Partial Resistance (PR)]; **B** DEGs also identified in the comparison within susceptible groups (MS against VS).

DEG analysis showed clear transcriptomic changes associated with fetal PRRS susceptibility (Additional file 5). Of the 500 most variable genes analyzed in the PCA, 85% (*N* = 426) were differentially expressed between fetal groups. We identified 3680 distinct DEGs from 8571 DEGs across each of the six pairwise comparisons between four fetal groups (CR, PR, VS, MS), with the larger sets of DEGs detected in the comparisons between MS fetuses and the resistant fetuses (CR, PR) (Table 2). Notably, no DEGs were identified between PR and CR fetuses, indicating a minimal transcriptional response to PRRSV infection in the PR group, similar to the CR fetuses. In contrast, 1165 DEGs distinguished MS fetuses from VS fetuses, highlighting transcriptional differences within the susceptible group that were consistent with the PCA results. Figure 3 visualizes the numbers of unique and shared DEGs across the comparisons. The Functional roles of thymic DEGs observed between resistant and susceptible fetuses were also investigated using GSEA. This analysis highlighted a consistent pattern across the comparisons between resistant and susceptible fetuses. All top 10 enriched pathways for upregulated genes in susceptible fetuses were related to immune response and signaling including interferon alpha/beta and gamma response pathways, along with interleukin 10 signaling and chemokine interactions (Additional files 6–9). However, pathways involved in cell cycle processes and DNA repair were enriched for downregulated genes in susceptible fetuses (Additional files 6–9). This enrichment was also observed in MS versus VS fetuses (Additional file 10). Top enriched pathways for upregulated genes in MS versus VS fetuses included pathways related to extracellular matrix (ECM) function (Additional file 10).

**Table 2:** Number of DEGs^*^ **from pairwise comparisons among the fetal groups**

Contrast^#^	Down-regulated	Up-regulated	Total
PR-CR	0	0	0
VS-CR	133	754	887
MS-CR	457	2264	2721
VS-PR	166	797	963
MS-PR	514	2321	2835
MS-VS	78	1087	1165

^*^ Differentially expressed genes, determined at False Discovery Rate (FDR) < 0.05 and log_2_ Fold Change (FC) < -1 (down-regulated) or log_2_ FC > 1 (up-regulated).

^#^Pairwise comparisons among the fetal groups; Complete Resistance (CR), Partial Resistance (PR), Viable Susceptible (VS), Meconium-stained Susceptible (MS). CR or PR served as the reference group when compared with VS or MS, and MS-VS denotes the comparison of MS versus VS (reference group).

**Figure 4:**
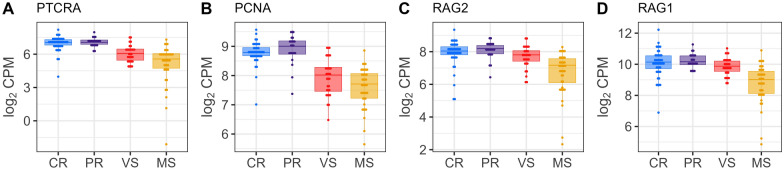
**Gene expression levels of DEGs encoding immature thymocyte markers in fetal thymus.** Immature thymocyte markers were identified in Gu et al.’s study [45]. Horizontal lines within the boxes indicate median log_2_ CPM values. Dots represent raw data overlaid on boxplots with fetal group differentiated by colors [Complete Resistance (CR), Partial Resistance (PR), Viable Susceptible (VS), Meconium-stained Susceptible (MS)]. Log_2_ counts-per-million (CPM) values were normalized by the TMM method and adjusted for fetal sex and batch.

**Figure 5:**
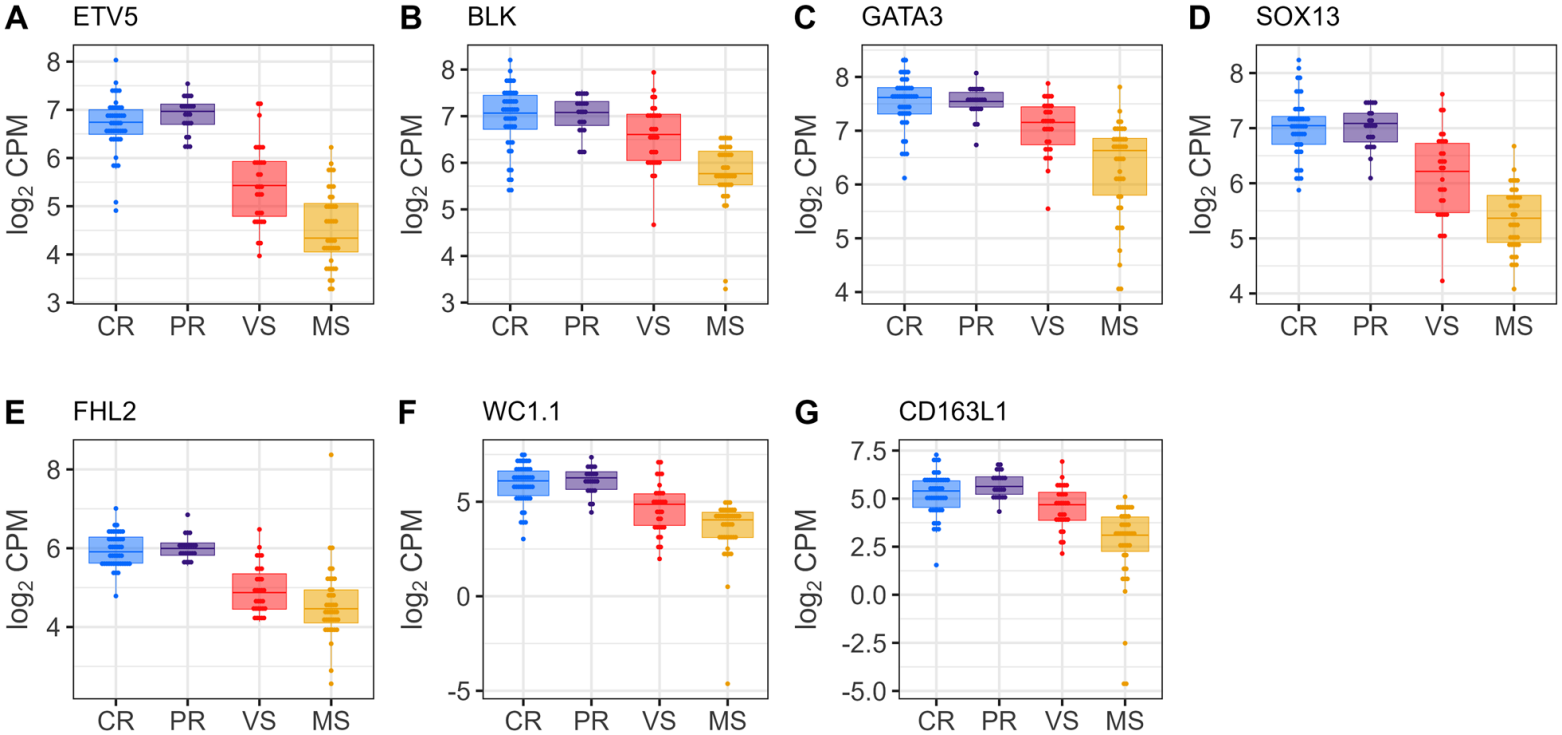
**Gene expression levels of DEGs encoding γδ T cell markers in fetal thymus.** γδ T cell markers were identified in Gu et al.’s study [45]. Horizontal lines within the boxes indicate median log_2_ CPM values. Dots represent raw data overlaid on boxplots with fetal group differentiated by colors [Complete Resistance (CR), Partial Resistance (PR), Viable Susceptible (VS), Meconium-stained Susceptible (MS)]. Log_2_ counts-per-million (CPM) values were normalized by the TMM method and adjusted for fetal sex and batch.

**Figure 6:**
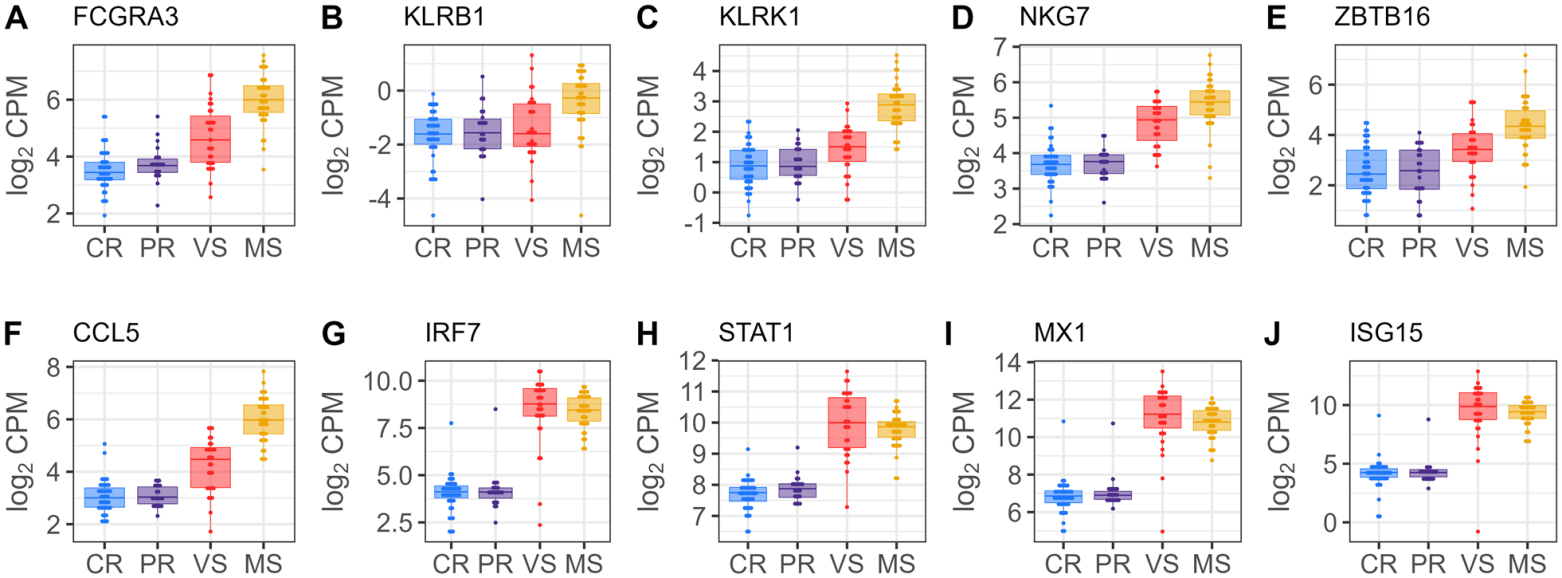
**Gene expression levels of DEGs encoding unconventional CD8+ cell markers in fetal thymus.** Unconventional CD8+ cell markers were identified in Gu et al.’s study [45]. Horizontal lines within the boxes indicate median log_2_ CPM values. Dots represent raw data overlaid on boxplots with fetal group differentiated by colors [Complete Resistance (CR), Partial Resistance (PR), Viable Susceptible (VS), Meconium-stained Susceptible (MS)]. Log_2_ counts-per-million (CPM) values were normalized by the TMM method and adjusted for fetal sex and batch.

**Figure 7:**
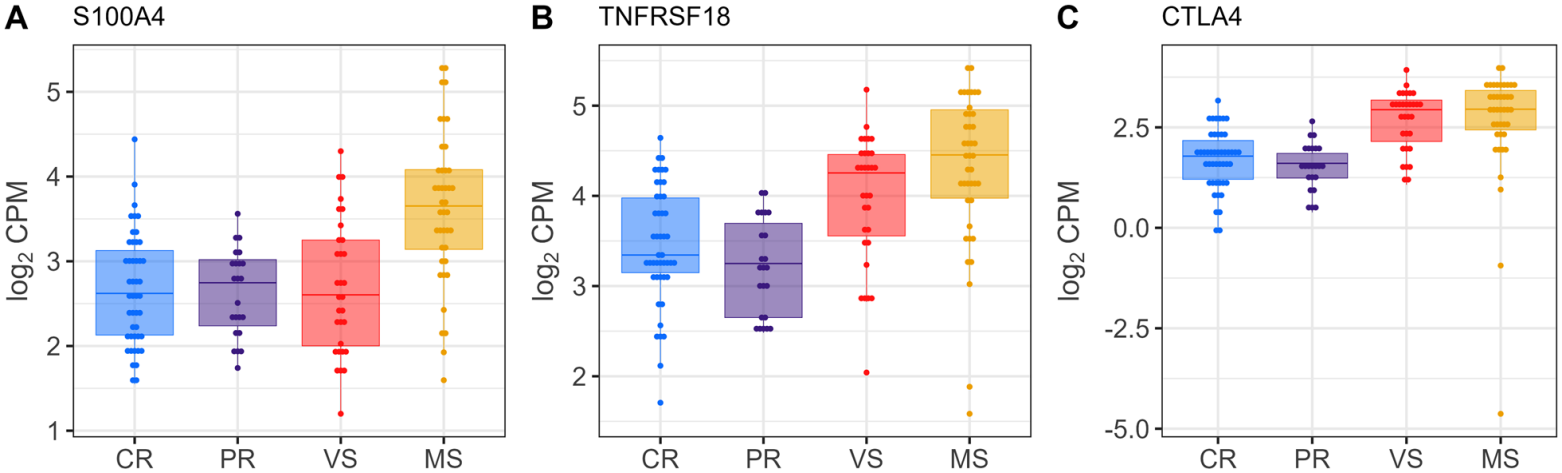
**Gene expression levels of DEGs encoding regulatory T cell markers in fetal thymus.** Regulatory T cell markers were identified in Gu et al.’s study [45]. Horizontal lines within the boxes indicate median log_2_ CPM values. Dots represent raw data overlaid on boxplots with fetal group differentiated by colors [Complete Resistance (CR), Partial Resistance (PR), Viable Susceptible (VS), Meconium-stained Susceptible (MS)]. Log_2_ counts-per-million (CPM) values were normalized by the TMM method and adjusted for fetal sex and batch.

Following the identification of DEGs, we explored their potential association with thymocyte development. We compared our DEGs (*n* = 3680) with two gene sets from the study of Gu et al. [45] (Additional file 11): (1) a set of thymocyte marker genes (*n* = 64) specific for various thymocyte subsets, and (2) a collection of 16 thymocyte module gene sets (*n* = 1448) representing distinct thymocyte development stages along the thymopoiesis pathway. This analysis revealed a significant overlap, with 444 DEGs matching thymocyte markers or genes within the thymocyte module sets associated with specific developmental stages for thymocytes. To further investigate these DEGs, we constructed individual box plots with dots depicting their expression levels across the fetal groups. This visualization identified a distinct transcriptional signature in the susceptible (MS) fetuses. Additional file 12 summarizes DEGs for thymocyte markers across Figures 4, 5, 6, 7, comparing fetal susceptibility groups (CR, PR, MS, VS) with contrasts VS-CR, VS-PR, MS-CR, MS-PR, and MS-VS. For double negative/double positive (DN/DP) thymocytes (Figure 4), key markers (*PTCRA*, *PCNA*, *RAG1*, *RAG2*), crucial for cell cycle progression and development of immature thymocytes (Gu et al. [45]), showed significant downregulation in MS versus CR/PR (e.g., *RAG1*, log_2_ FC = −1.127, FDR = 2.02E−07, MS-CR). Similarly, CD2−γδ T cell markers (e.g., *ETV5*, *BLK*, *WC1.1*; Figure 5) were downregulated in MS (e.g., ETV5, log_2_ FC = −2.039, FDR = 2.15E−20, MS-CR) and some VS comparisons. In contrast, unconventional CD8+ T cell (ISG-CD8 T, cytotoxic CD8 T, CD8αα cells) markers (e.g., *CCL5*, *IRF7*, *ISG15*; Figure 6) and regulatory T cell (Treg) markers (e.g., *CTLA4*, *S100A4*; Figure 7) were significantly upregulated in VS and MS versus CR/PR (e.g., ISG15, log_2_ FC = 5.141, FDR = 1.33E−28, MS-CR). The overlap between DEGs and those in the thymocyte module gene sets further supported the observed expression patterns. Specifically, downregulated DEGs were predominantly mapped to genes for immature thymocyte stages (double negative and double positive) and γδ T cells, whereas upregulated DEGs were mainly found within gene sets for post-committed thymocyte stages. To gain deeper insights into gene set contributions, we employed GESECA with thymocyte module gene sets. This approach also enabled us to visualize the co-regulation profiles of all thymically expressed genes within each module in a single plot (Additional file 13), providing a comprehensive overview of these pathways across fetal pigs. From this analysis, we identified two key gene sets that varied markedly in their expression across fetuses, corresponding to proliferating double positive thymocytes (modules 4, 5) and ISG-CD8 T cells (module 8) (Additional file 14). These gene sets exhibited significant coordinated expression changes across fetuses, differentiating susceptible group from resistant group (Additional file 13). Notably, the coregulation profile of genes for ISG-CD8 T cells mirrored the upregulation of interferon response genes in the susceptible group (Additional file 4). GSEA revealed significant enrichment of thymocyte module gene sets in susceptible versus resistant groups (Additional file 15). Post-committed thymocyte gene sets (modules 7–11) were enriched among upregulated genes. Conversely, immature thymocyte gene sets (modules 1–6) and gene sets for γδ T cells (modules 12–13) were enriched among downregulated genes. These results were consistent with the coregulated patterns observed in GESECA results (Additional file 13). Additionally, the ISG-CD8 T cell gene set (module 8) enriched for ISGs showed enrichment for downregulated genes in MS compared to VS fetuses (Additional file 15) but enriched for upregulated genes when comparing susceptible to resistant fetuses. Key genes contributing to the enrichment of this module in the MS versus VS comparison included *ENSSSCG00000033089*, *SAMD9*, *EPSTI1*, *EIF2AK2*, *PARP14*, *GHSR*, *ISG15*, *DDX60*, *IFIH1*, *DHX58*, *MX1*, *RSAD2*, *IFI44*, *HERC5*, *MX2*, *IFIT1*, although these genes were not differentially expressed in MS versus VS fetuses.

The main transcriptional differences that differentiate resistant fetuses (CR, PR) from susceptible fetuses (VS, MS) are summarized in Table 3.

**Table 3:** **Summary of main transcriptional differences between resistant (CR, PR) and susceptible (VS, MS) fetuses**

Fetal group^*^	Gene expression changes: MSigDB gene sets (human)	Gene expression changes: pig thymocyte gene sets^#^
CR	Reference group for DEG^**^	Reference group for DEG
PR	no DEG	no DEG
VS	• Upregulation of interferon response genes • Downregulation of cell cycle genes	• Downregulation of genes involved in early thymocyte development • Upregulation of genes associated with mature thymocytes and regulatory T cells
MS	Similar to VS but with additional upregulation of extracellular matrix genes when compared with VS	Further downregulation of genes involved in early thymocyte development compared to VS

^*^Complete resistance (CR), partial resistance (PR), viable susceptible (VS), meconium-stained susceptible (MS).

^#^Identified in Gu et al.’s study [45].

^**^Differentially expressed genes, determined at False Discovery Rate (FDR) < 0.05 and log_2_ Fold Change (FC) < −1 (down-regulated) or log_2_ FC > 1 (up-regulated).

### Genome-wide detection of interaction eQTLs for DEGs

We examined the interaction between genotypes and fetal groups on the expression levels of differentially expressed genes (DEGs), hypothesizing that genotype variation influences gene expression differently depending on fetal PRRS susceptibility. Using a linear mixed effects model, we tested this interaction to detect loci exhibiting divergent effects (e.g., opposing directions of expression change) or varying magnitudes (e.g., stronger effects in one susceptibility group) between fetal groups, thereby sought to link context-specific genetic effects to differential PRRS susceptibility. This statistical interaction does not imply a direct molecular interaction but rather a differential genetic effect under distinct PRRS disease conditions represented by fetal groups. This interaction eQTL mapping analysis tested genotype-by-fetal group interactions for 129 469 unique genetic variants including 60 K SNPs and RNA-seq derived variants (43 113 SNPs from 60 K SNP data and 74 481 SNPs and 12 546 indels from RNA-seq data; among a total of 130 140 DNA variants, 661 SNPs were shared between the two SNP datasets, and 10 loci for RNA-seq indels were shared with 60 K SNP loci) across four fetal groups, focusing on 3680 DEGs. We identified 108 unique cis-acting SNPs or indels (cis-ieQTLs) linked to the expression of 57 DEGs (termed ieGenes), resulting in 125 distinct genetic associations that varied by fetal group (Additional file 16). The majority (62%) of the 108 cis-eQTLs reported are within 500 kb, consistent with pig specific linkage disequilibrium decay (100–500 kb, with average r^2^ of 0.19–0.26 at 500 kb and 0.15–0.20 at 1 Mb across four US pig breeds) [53]. In detail, the distribution was 15 (13.9%) within 100 kb, 26 (24.1%) in the 100–250 kb range, 26 (24.1%) in the 250–500 kb range, with 41 (38%) in the 500 kb–1 Mb range.

The ieGenes were comprised of 55 protein-coding genes, 1 lncRNA gene (*ENSSSCG00000055788*) and 1 snoRNA gene (*SNORA80E*). ieGenes (*N* = 27) were annotated with the curated gene sets that were significantly associated with thymic gene expression variance (Additional file 17). Some genes were involved in multiple significant pathways, such as *CXCL10* (*N* = 13), *CXCL9* (*N* = 10), *CCR10* (*N* = 9), *CXCL11* (*N* = 9), *COL15A1* (*N* = 9), *DYNC1I1* (*N* = 9), *SPHK1* (*N* = 8), *SCN2B* (*N* = 7). *CCR10*, *CXCL10*, *CXCL11*, and *CXCL9* were grouped together within the pathways related to chemokines or G protein-coupled receptors (GPCRs). Among the ieGenes, 9 genes were identified in the thymocyte module gene sets: *ATF3* (module 1), *PTCRA* (module 1), *ENSSSCG00000030801* (*LOC102161784*; Guanylate-binding protein 6-like; module 2), *MNDA* (module 6), *SERPINB10* (module 7), *DHX58* (module 8), *NR4A3* (module 9), *PMAIP1* (module 11), *ENSSSCG00000000640* (the pig orthologue of the human *KLRC1*; module 11).

**Figure 8:**
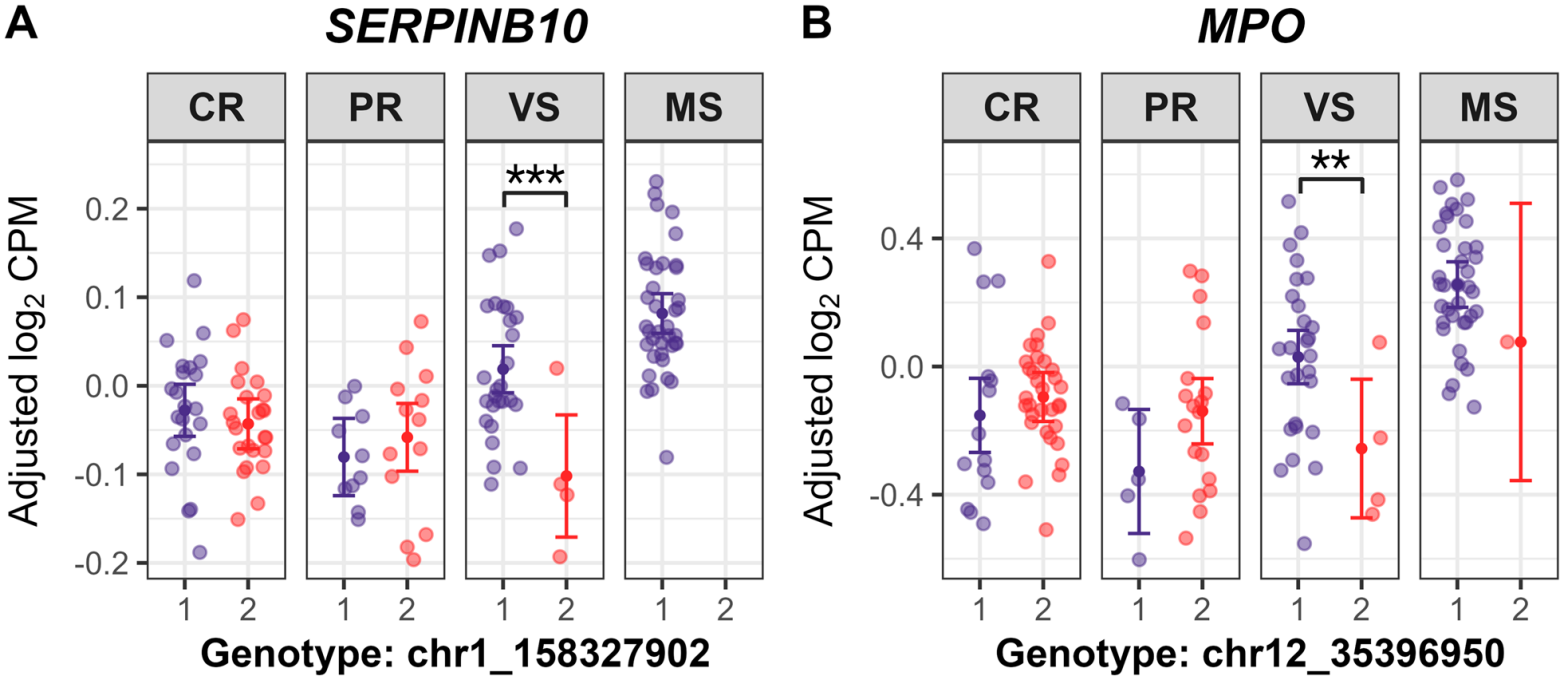
**Estimated marginal means on expression levels of SERPINB10 and MPO by genotype and fetal group**. chr1_158327902 (**A**), chr12_35396950 (**B**) indicate SNP names for sequence variants not reported in VEP annotations (Ensembl release-110). **A**, **B** Genotype 1, heterozygous; genotype 2, homozygous for variant allele in the genotype data used for the study. Large central dots and associated vertical lines represent the estimated marginal means (EMM) and 95% confidence interval, respectively. Overlapping small dots are the conditional residuals of log_2_ CPM values from the null eQTL model adjusting for genetic relatedness among fetal pigs. *P* value adjustment was conducted using the Benjamini-Hochberg (BH) method for all tests within genotype and within fetal group. BH-adjusted *P* < 0.05 are labeled for the significant pairwise comparisons of the EMMs within fetal group; *P* < 0.0001, ***; 0.0001 ≤ *P* < 0.01, **. Genotype counts (genotype 0/1/2): **A** *SERPINB10*, CR: 0/22/24, PR: 0/10/13, VS: 0/27/4, MS: 0/38/0; **B** *MPO*, CR: 0/14/32, PR: 0/5/18, VS: 0/27/4, MS: 0/37/1.

**Figure 9:**
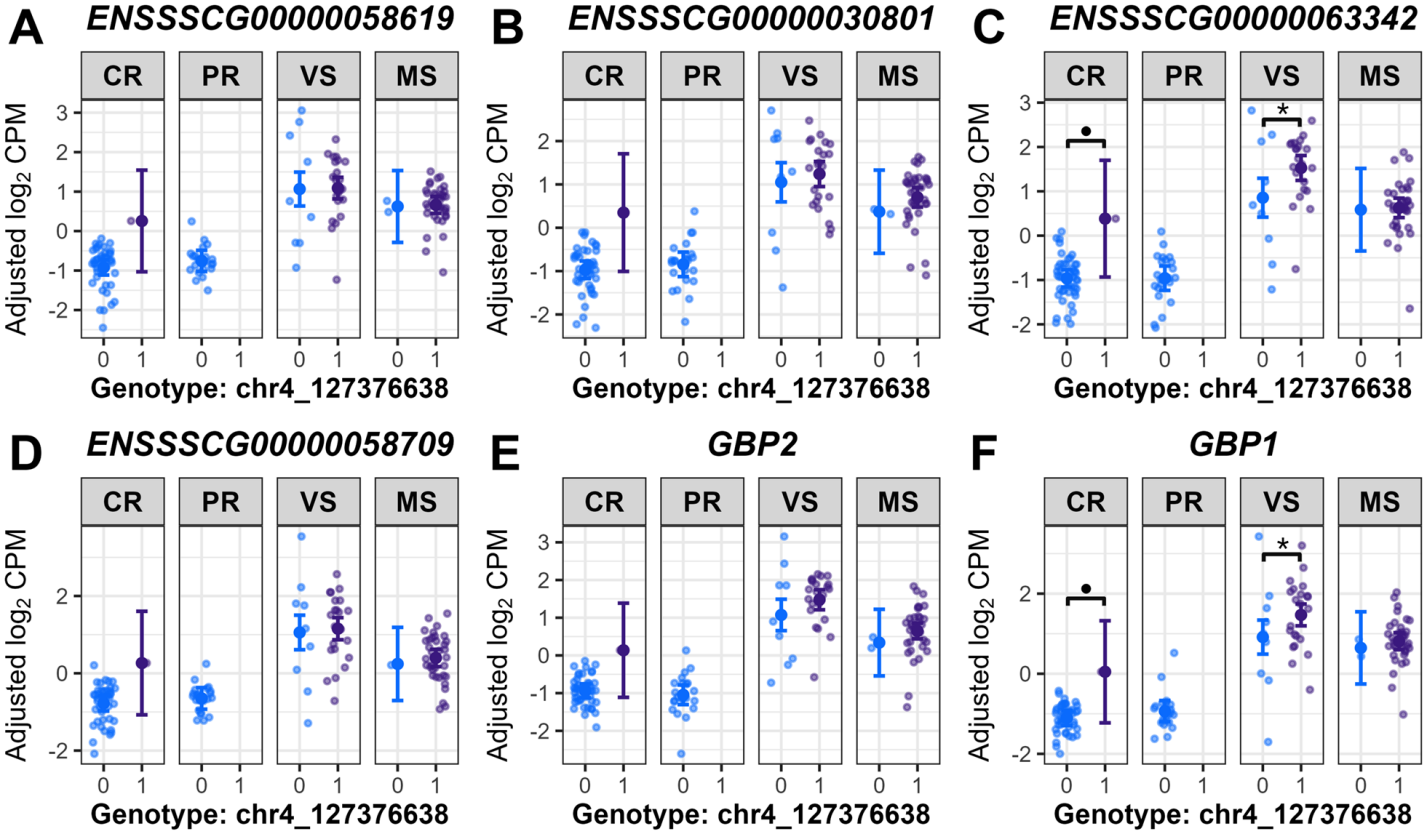
**Estimated marginal means on expression levels of GBP family genes by genotype and fetal group.** **A**–**F** Genes are listed in order of their chromosomal position; Gene annotations from the NCBI Gene Database for four guanylate-binding protein (GBP) genes; **A** *ENSSSCG00000058619*, *LOC100523310* (*guanylate-binding protein 6*); **B** *ENSSSCG00000030801*, *LOC102161784* (*guanylate-binding protein 6-like*); **C** *ENSSSCG00000063342*, *LOC100523668* (*guanylate-binding protein 2-like*); **D** *ENSSSCG00000058709*, *LOC100155195* (*guanylate-binding protein 7*). **A**–**F** chr4_127376638, SNP name for a sequence variant not reported in VEP annotations (Ensembl release-110). Genotype 0, homozygous genotype for reference allele; genotype 1, heterozygous in the genotype data used for the study. Large central dots and associated vertical lines represent the estimated marginal means (EMM) and 95% confidence interval, respectively. Overlapping small dots are the conditional residuals of log_2_ CPM values from the null eQTL model adjusting for genetic relatedness among fetal pigs. *P* value adjustment was conducted using the Benjamini-Hochberg (BH) method for all tests within genotype and within fetal group. BH-adjusted P values are labeled for significance or a trend in pairwise comparisons of the EMMs within fetal group; 0.01 ≤ *P* < 0.05, *; 0.05 ≤ *P* < 0.10, •. Genotype counts (genotype 0/1/2) (**A**–**F**): CR: 45/1/0, PR: 23/0/0, VS: 9/22/0, MS: 2/36/0.

**Figure 10:**
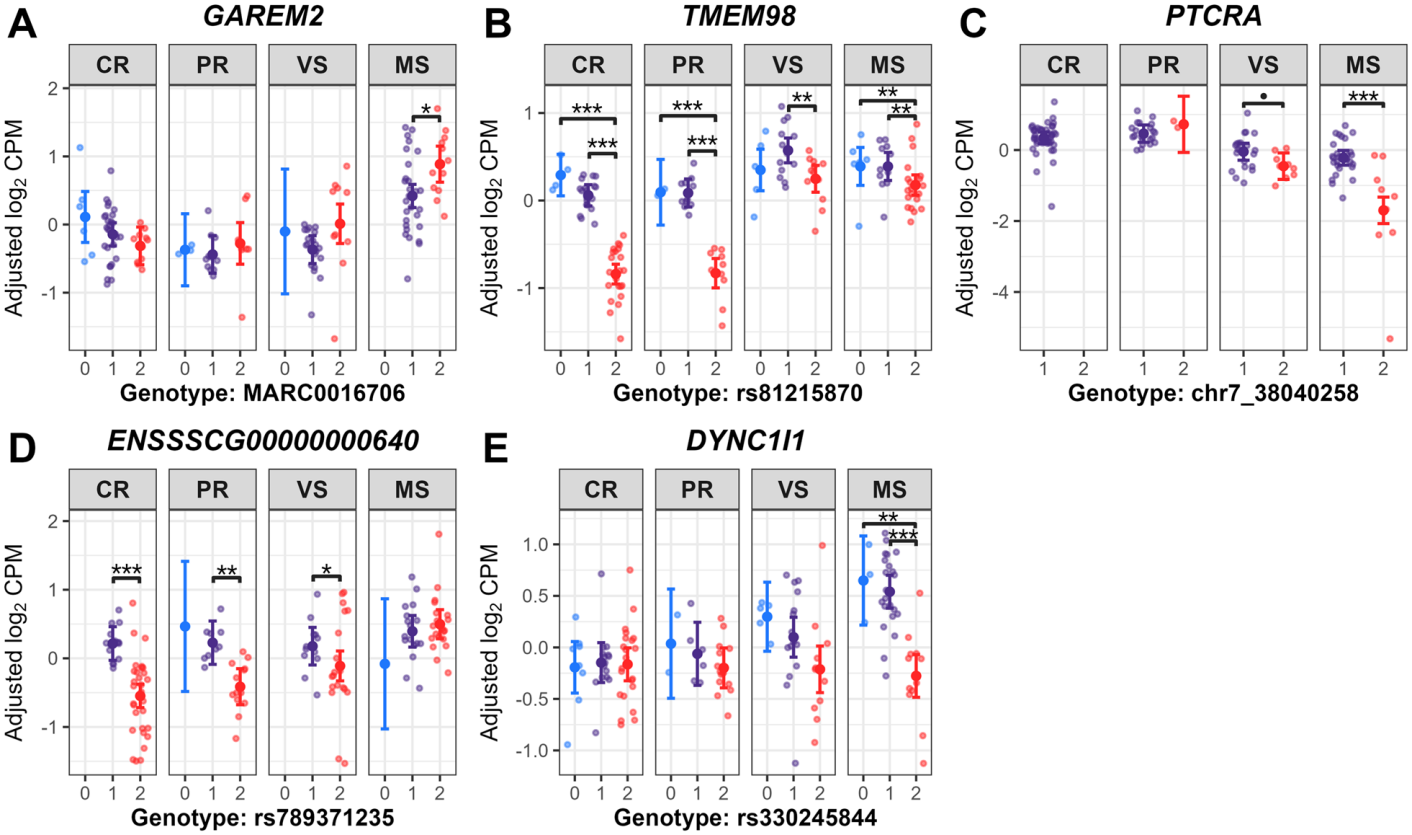
**EMMs on expression levels of GAREM2, *TMEM98, PTCRA*, *ENSSSCG00000000640*, *DYNC1I1 by genotype and fetal group.*** **A** MARC0016706, PorcineSNP60 v2 genotyping Beadchip ID (rs81285919, dbSNP ID); **B** rs81215870, dbSNP ID; **C** chr7_38040258, indel name for a sequence variant not reported in VEP annotations (Ensembl release-110); **D** rs789371235 (indel), dbSNP ID. Gene annotation from the NCBI Gene Database for *ENSSSCG00000000640* was orthologous to human *KLRC1*; **E** rs330245844 dbSNP ID. **A**–**E** Genotype 0, homozygous genotype for reference allele; genotype 1, heterozygous; genotype 2, homozygous for variant allele in the genotype data used for the study. Large central dots and associated vertical lines represent the estimated marginal means (EMM) and 95% confidence interval, respectively. Overlapping small dots are the conditional residuals of log_2_ CPM values from the null eQTL model adjusting for genetic relatedness among fetal pigs. *P* value adjustment was conducted using the Benjamini-Hochberg (BH) method for all tests within genotype and within fetal group. BH-adjusted *P* values are labeled for significance or a trend in pairwise comparisons of the EMMs within fetal group. *P* < 0.0001, ***; 0.0001 ≤ *P* < 0.01, **; 0.01 ≤ *P* < 0.05, *; 0.05 ≤ *P* < 0.10, •. Genotype counts (genotype 0/1/2): **A** *GAREM2*, CR: 6/29/11, PR: 3/11/9, VS: 1/20/10, MS: 0/29/12; **B** *TMEM98*, CR: 5/19/22, PR: 2/11/10, VS: 5/14/12, MS: 6/11/21; **C** *PTCRA*, CR: 0/46/0, PR: 0/21/2, VS: 0/22/9, MS: 0/29/9; **D** *ENSSSCG00000000640*, CR: 0/15/31, PR: 1/9/13, VS: 0/12/19, MS: 1/17/20; **E** *DYNC1I1*, CR: 9/15/22, PR: 2/6/15, VS: 5/15/11, MS: 3/22/13.

Post-GWAS analysis, including estimated marginal means (EMMs) analyses, was used to quantify genotype-by-fetal group associations for selected ieQTL variants, estimating expression means (adjusted log_2_ CPM) for genotype-fetal group combinations of selected ieGenes. This analysis revealed genotype-driven expression differences due to ieQTL interactions, indicating genotype’s effect on expression is not constant across fetal groups. Two genes involved in neutrophil degranulation (Additional file 17), *SERPINB10* and *MPO*, were upregulated in VS or MS fetuses across all DEG contrasts. *SERPINB10* (associated with the chr1_158327902 variant) and *MPO* (associated with the chr12_35396950 variant) both showed significantly lower expression in VS fetuses that were homozygous for the variant allele, compared to those with the heterozygous genotype (*P* = 7.21E−6 for *SERPINB10*, *P* = 5.75E−4 for *MPO*) (Figure 8). The genotype-by-fetal group interactions for these ieQTLs may have been driven by a bias in genotype proportions, with most VS fetuses (27/31, ~87%) heterozygous, and MS fetuses showing even numerically higher heterozygous frequencies (100% for *SERPINB10*; 1/38, ~97% for *MPO*). Conversely, CR and PR fetuses showed numerically higher frequencies for the homozygous genotypes (52.2–56.5% for *SERPINB10*; 69.6–78.3% for *MPO*). Hence, this skewed distribution between susceptible (VS/MS) and resistant (CR/PR) groups may affect the detectability of ieQTL interactions, as heterozygous genotypes in VS/MS were associated with elevated *SERPINB10* and *MPO* expression. A single SNP (chr4_127376638) interacted significantly with fetal groups, being associated with the expression of six ieGenes clustered within the guanylate-binding proteins (GBPs) gene family region (Figure 9). This region is known as PRRS disease resistance QTL in young piglets [55–58]. In VS fetuses, higher expression levels of *ENSSSCG00000063342* (*LOC100523668*, Guanylate-binding protein 2-like) and *GBP1* were observed in the heterozygous genotype versus homozygous genotype for the reference allele (*P* = 0.012 for *ENSSSCG00000063342*, *P* = 0.015 for *GBP1*) (Figures 9C, F). The variant allele of chr4_127376638 was more prevalent in susceptible versus resistant groups, with genotype counts showing numerically higher heterozygous frequencies in VS (71%) and MS (94.7%) compared to CR (2.2%) and PR (0%), where resistant fetuses (except one CR fetus) were homozygous for the reference allele. This SNP was found in the 3ʹ UTR of *ENSSSCG00000058709* (*LOC100155195*, Guanylate-binding protein 7) or downstream region of *GBP2* within the GBP gene cluster. Only one cis-acting SNP was detected from the eQTL mapping using the 60 K SNPs. Expression level of *GAREM2* was significantly associated with a SNP (MARC0016706; rs81285919) whose effect varied by fetal group. Homozygous genotype for the variant allele was associated with higher *GAREM2* expression compared to heterozygous genotype in MS fetuses (*P* = 0.004), whereas this effect was not significant in other fetal groups (Figure 10A). Notably, expression level of *TMEM98* was associated with two ieSNPs in the 5ʹ UTR (rs320867996; c.−26C>T) and the coding region (rs81215870; p.Ser60=, synonymous variant), respectively. These two SNPs were highly correlated based on the linkage disequilibrium (r^2^ = 0.903). Lower gene expression level was observed in homozygous genotype for the variant allele of the rs81215870 SNP compared to the other two genotypes in CR, PR, and MS fetuses (*P* < 0.01 for all comparisons; Figure 10B). Gene expression level was significantly lower in the homozygous genotype for the variant allele versus the heterozygous genotype within MS fetuses for *PTCRA* (*P* = 3.63E−10) and *DYNC1I1* (*P* = 6.24E−8) (Figures 10C, E). One of the thymocyte module genes, *ENSSSCG00000000640* (orthologous to human *KLRC1*), exhibited significantly higher gene expression in the heterozygous genotype compared to the homozygous genotype for the variant allele in the CR (*P* = 5.12E−8), PR (*P* = 0.005), and VS (*P* = 0.04) groups (Figure 10D). Additionally, no positional candidate genes for ieQTLs were overlapped with candidate genes for fetal PRRS disease phenotype QTLs reported in the GWAS of Yang et al. [17] and Van Goor et al. [18].

## Discussion

### Dysregulated thymic T cell development is associated with increased fetal susceptibility to PRRSV

PRRSV infection has a detrimental impact on the developing porcine thymus [14, 59]. Specifically, the virus targets CD14+ antigen-presenting cells in the thymus, leading to indirect apoptosis of double positive thymocytes [14]. This, along with reduced prothymocyte production in the bone marrow and PRRSV-induced autophagy in thymic epithelial cells [14], can disrupt normal T cell development and selection, potentially leading to increased PRRS susceptibility. Our transcriptomic analysis, comparing DEGs with established thymocyte marker genes from the published datasets from Gu et al. [45], revealed a transcriptional signature indicative of disruptions in the early stages of T cell development within the severely PRRSV-infected fetal thymus, along with upregulation in mature thymocyte marker gene expression. GESECA and GSEA further suggested that susceptible fetuses display shifts in thymocyte developmental profiles compared to resistant fetuses. Genes crucial for early-stage thymocyte development were downregulated, while those associated with more mature thymocytes were upregulated. Thus, these results may indicate an accelerated progression of T cell development within the PRRSV-infected fetal thymus, which may compromise the maturation process and increase susceptibility to PRRSV. In the previous transcriptome study using the same pregnant gilt PRRSV-2 challenge model, Wilkinson et al. [7] identified gene expression patterns suggestive of an excessive inflammatory state in meconium-stained (MEC) fetuses. They hypothesized that this response could potentially damage thymic tissue, leading to a reduction in T cells within the thymus of these fetuses. This hypothesis is supported by subsequent observations by Malgarin et al. [2] who found apoptotic cells concentrated in the cortex and corticomedullary border of the thymus in highly infected fetuses, including MEC fetuses. Since these areas are a crucial thymic microenvironment enriched for developing thymocytes at early stages [9], transcriptional dysregulation of thymocyte development observed in our study provides further support for Wilkinson et al.’s hypothesis [7], Butler et al.'s hypothesis of PRRSV-induced thymic dysfunction [59], and the recent finding that demonstrated the direct effect of PRRSV on thymocytes causing a diminished pool of double positive thymocytes within the thymic cortex near the corticomedullary borders [15]. The downregulation of CD2− γδ T cell marker DEGs also support this observation since these cells were shown to be differentiated from double negative thymocytes [45]. While mRNA expression profiles of various thymocyte markers were examined in this study, it is important to note that some of these markers may not be exclusively specific to thymocyte subsets. For instance, *PCNA*, involved in cell cycle progression, is not thymocyte-specific and could be expressed in other proliferating cells [60] or even in non-proliferating cells such as neutrophils [61]. Therefore, the interpretation of these markers should be considered in the context of a broader set of thymocyte markers rather than relying on a single marker, as this offers a more comprehensive understanding of the different stages involved in thymocyte development.

Meanwhile, we also observed upregulation of markers for unconventional CD8+ T cells, including ISG-CD8 T cells and cytotoxic CD8 T cells, and regulatory T cells in susceptible versus resistant fetuses. The ubiquitous expression of genes across these thymocytes and other thymic immune cells may contribute to the observed changes in thymic gene expression. For instance, some of the marker genes for cytotoxic CD8 T cells or CD8αα cells, such as *KLRB1*, *XCL1*, *NKG7*, etc.*,* have been also found to be expressed in natural killer (NK) cells in the human thymus [62]. Furthermore, these unconventional CD8+ T cell populations have a similar gene expression profile with pig invariant natural killer T (iNKT) cell subsets [45]. While local iNKT cell response could also contribute to this observation, their generally low frequency in the thymus [63] suggests additional thymic cell response may be involved. One possibility is that PRRSV infection activates intrathymic T cells through enhanced cytokine expression, including cytokines known to influence CD8+ T cell differentiation or regulatory T cell development [9]. Indeed, GSEA for biological pathways revealed a significant enrichment for cytokine pathways at the top ranked upregulated genes in susceptible fetuses versus resistant fetuses. For instance, genes directly known to code for cytokines (*IFNB1*, *IFNG*) or closely associated with the regulation or modulation of cytokine functions such as interferon regulatory factors (*IRF1*, *IRF5*, *IRF7*, etc.) [64] were identified in the interferon response gene sets enriched for upregulated genes in susceptible fetuses versus resistant fetuses. Genes induced by interferons (*MX1*, *MX2*, *ISG15*, etc.) [65] were also found to be upregulated in susceptible versus resistant fetuses. Furthermore, increased immunohistochemical expression of cytokines, such as IFN-γ and TNF-α, and FOXP3, a transcription factor involved in regulatory T cell differentiation [66], has been identified in the thymic medulla of piglets infected with the virulent PRRSV-1 strain [13]. Since activated peripheral T cells do not recirculate to the thymus [16], the observed alterations in expressions of genes associated with markers and DEGs specific to thymocyte development likely arise from PRRSV’s direct impact within the fetal thymus.

Taken together, our data suggests a potential association between the altered thymic T cell development and fetal PRRS susceptibility, although the directionality of this relationship remains unclear. For instance, there is another possible scenario of pre-existing fetal vulnerability underlying these findings, where susceptible fetuses may have already had differential T cell dynamics prior to PRRSV infection, possibly limiting their ability to effectively control viral replication. Thus, further investigation is warranted to determine whether the altered thymic T cell development is the outcome from the causal effect of PRRSV infection or not. In either scenario, our findings indicate the potential relationship between PRRSV infection and fetal thymic function.

The observed changes in gene expression in the current study strongly support the idea of a localized response to PRRSV infection in fetuses [12], with the most pronounced changes in gene expression observed in the MS group, as evidenced by their larger set of DEGs compared to the VS group. We hypothesized that the compartmentalized fetal response to PRRSV may be affected by neighboring fetal conditions via horizontal PRRSV transmission, based on the observation of the in utero pattern of clusters of infected and dead fetuses [21], where the presence of neighboring fetuses that were either infected with PRRSV or dead significantly increased the likelihood of fetal death [22]. Thus, we aimed to account for the possible effect of neighboring fetuses on fetal response by defining resistant and susceptible fetuses based on the infection status of adjacent fetuses as described in Figure 1. For instance, an uninfected viable fetus with 2 dead neighboring fetuses was considered resistant, but a highly infected viable fetus with 2 neighboring uninfected fetuses was susceptible. However, the main transcriptional response between resistant and susceptible fetuses resembles previous findings [3–5], where neighboring fetal condition was not considered when selecting fetuses. Susceptible fetuses (VS, MS group) exhibited immune activation while suppressing cellular growth and transcription compared to resistant fetuses (CR, PR group), which was observed in infected fetuses (viable and high serum and thymic viral load) versus uninfected fetuses (viable with no virus detected in fetal serum and/or thymus) in these previous studies [3–5]. While the clustering of infected or dead fetuses suggests that a neighboring fetal infection effect could influence fetal outcomes, our findings indicate that, if it exists, it is not a primary determinant of the major changes in thymic gene expression observed in PRRSV-infected fetuses. The small sample size in our ieQTL analysis, due to limited fetal thymic samples, may have reduced statistical power and likely contributed to fixed alleles in some groups (Figures 8 and 9). Despite this, our study focused on thymic transcriptomic variation across different fetal susceptibility levels, considering potential neighboring fetal effects. Future studies with larger cohorts or targeted resistant-versus-susceptible ieQTL analyses could validate these findings and deepen our understanding of PRRS susceptibility in fetal pigs.

We also sought to identify which thymocyte gene sets or functional gene sets best distinguish the MS group from the VS group to gain insight into fetal susceptibility mechanisms. MS fetuses showed significant upregulation of extracellular matrix (ECM) associated genes [67], such as collagen related genes (e.g., *COL21A1*, *COL12A1*, *COLQ,* etc.), integrin genes (e.g., *ITGA11*, *ITGAM*, *ITGA7*, etc.), fibronectin associated genes (e.g., *FNDC4*, *FLRT2*, *FSD2*, *EGFLAM*, etc.), and laminin genes (*LAMC3*, *LAMB2*, *LAMC1*, *EGFLAM*, *LAMA5*), compared to VS fetuses. Functional enrichment analysis also showed that genes relevant to ECM functions collectively contributed to the significant enrichments of “extracellular matrix organization”, “integrin1 pathway”, “MET activates PTK2 signaling”, “UPA-UPAR pathway”, and “focal adhesion” among the enriched pathways for upregulated genes in MS versus VS fetuses. Specifically, ECM genes may originate from nonimmune cells such as fibroblast type 2 (Fb2) cells which were found to be localized in interlobular space in the human thymus, expressing these genes [62]. Based on the study of Park et al. [62], human thymic Fb2 cells have been also shown to express genes associated with vascular development. Collectively, these data suggest that upregulations in ECM-related genes in MS fetuses may reflect either a disruption of thymic architecture triggered by severe PRRSV infection or differences in thymus vascularization related to increased fetal susceptibility. Dysregulated ECM genes could also indicate altered interactions between thymocytes and ECM molecules, potentially impeding thymopoiesis [66]. Overall, these findings highlight that PRRSV-associated disruptions involving nonimmune thymic cells likely contribute to the distinct gene expression profiles observed in MS versus VS fetuses, with the MS group showing more pronounced changes. This supports a connection between severe PRRSV infection, ECM dysregulation, and the potential impairment of thymopoiesis. Meanwhile, the most significantly enriched pathways for downregulated genes in MS versus VS fetuses were similar to those of other contrasts (i.e., susceptible versus resistant groups), where cell cycle and mRNA processing pathways were downregulated. Viruses can induce widespread degradation of host mRNA, using virally encoded proteins functioning as endonucleases or decapping enzymes, in order to prioritize their own replication [68, 69]. Thus, the downregulation of host mRNA processing pathways observed in MS fetuses compared to VS fetuses likely suggests a more extensive and/or severe disruption of host mRNA production and stability, potentially caused by thymic PRRSV infection.

### The thymic ieQTLs focusing on TMEM98 involved in T helper 1 cell differentiation

None of 108 unique cis-ieQTLs linked to the expression of 57 DEGs clearly differentiated fetal susceptibility groups, however, our findings indicate that fetal genotypes can differentially influence thymic gene expression depending on the level of fetal PRRS susceptibility. We prioritized *TMEM98* due to its biological relevance to thymic function, particularly its roles in Th1 cell differentiation [70] and IL-8-mediated vascular inflammation [71], which may be pertinent to PRRSV immunopathology. We identified two ieQTLs in *TMEM98* showing genotype-dependent regulation of *TMEM98* expression within each fetal group. Specifically, the CC genotype (genotype 2) of the 5’ UTR SNP showed significantly lower *TMEM98* expression than the heterozygous CT genotype (genotype 1) in each fetal group. This suggests the variant modulates expression magnitude rather than directly driving differential susceptibility.

This gene encodes the type II transmembrane protein 98 (TMEM98), which is expressed on the cell surface or secreted through exosomes [70]. Importantly, TMEM98 has been identified as a potential cytokine that promotes the differentiation of naïve CD4+ T cells into T helper 1 (Th1) cells [70] which activate macrophages and cytotoxic T lymphocytes (CTLs) [72]. Based on TMEM98’s role in promoting Th1 differentiation [70], the observed increase in *TMEM98* expression in susceptible fetal pigs versus resistant ones may indicate an association with enhanced Th1 cell differentiation and a stronger Th1-mediated proinflammatory response against intracellular PRRSV antigens [73]. Furthermore, genotypic differences in *TMEM98* expression within each fetal group (e.g., higher expression in the heterozygous genotype versus the homozygous variant genotype) may indicate genetic factors predispose fetal pigs to varying extents of Th1 response. Further functional studies are necessary to determine whether the elevated *TMEM98* expression and associated Th1 response confer a protective advantage or exacerbate disease severity in the context of PRRSV infection.

Besides the effect on CD4+ T cell differentiation, TMEM98 may be involved in immune cell recruitment to the vessel wall and in the proliferation and migration of vascular smooth muscle cells (VSMCs) in response to the pro-inflammatory cytokine, IL-8 (i.e., CXCL8) [71]. *TMEM98* expression is upregulated by IL-8 in blood vessel wall cell lines (endothelial cells (ECs) or VSMCs) [71]. Knockdown of *TMEM98* using siRNA has been demonstrated to reduce monocyte adhesion to ECs by downregulating the expression of the ICAM-1 in the ECs, and to inhibit VSMC proliferation and migration in part by suppressing the AKT/GSK3β/Cyclin D1 signaling pathway [71]. Conversely, *TMEM98* overexpression increased basal levels of VSMC proliferation and migration [71]. Thus, increased *TMEM98* expression in susceptible fetal pigs may be interpreted as a sign of heightened inflammation (vasculopathy) in the cortico-medullary venules permeable to circulating foreign antigens or at the blood-thymus barrier with increased permeability [74], a response triggered by PRRSV infection. When combined with the genotype-dependent effects on *TMEM98* expression, these findings may also indicate inherent differences by genotype in the thymic vasculature as a pre-existing vulnerability factor in susceptible fetal pigs. Further research is needed to establish a causal relationship among *TMEM98* variants, *TMEM98* expression and its role in Th1 response and vascular alterations, and fetal susceptibility to PRRSV.

We provide transcriptional evidence that severe PRRSV infection is linked to disruption in normal T cell development within the fetal thymus. It is proposed that thymic DEGs encoding developing thymocyte markers show consistent downregulation in susceptible fetuses (VS, MS) compared to resistant fetuses (CR, PR). Such DEGs may collectively serve as transcriptional markers to predict fetal PRRS susceptibility through a distinct expression signature. Fetuses with downregulated early thymocyte development markers (e.g., *PTCRA*, *RAG1*, *ETV5*) and upregulated unconventional CD8+ T cell markers (e.g., *CCL5*, *FCGRA3*, *KLRB1*) are highly likely to be susceptible (VS, MS). We also provide evidence that a fetal genetic component to the thymic transcriptional changes may be an important factor explaining the variation in the severity of fetal PRRS disease. Collectively, these integrated transcriptomic and genomic analyses underscore the complexity of thymic transcriptional alterations related to fetal genotypes following PRRSV infection in fetal pigs at late gestation.

## Supplementary Information

**Additional file 1. Top 20 hallmark gene sets significantly associated with variability in fetal thymic transcriptome.**

**Additional file 2. Top 20 REACTOME and KEGG pathways from the curated gene sets significantly associated with variability in fetal thymic transcriptome.**

**Additional file 3. Complete gene lists for top 20 hallmark gene sets and REACTOME and KEGG pathways significantly associated with variability in fetal thymic transcriptome.**

**Additional file 4. Co-regulation profiles of genes within the top 3 hallmark gene sets significantly associated with variability in fetal thymic transcriptome.**

**Additional file 5. Gene expression changes in each of the six pairwise comparisons between four fetal groups (CR, PR, VS, MS).**

**Additional file 6. Top 10 pathways showing significant enrichment in either upregulated or downregulated genes in VS versus CR group.**

**Additional file 7. Top 10 pathways showing significant enrichment in either upregulated or downregulated genes in MS versus CR group.**

**Additional file 8. Top 10 pathways showing significant enrichment in either upregulated or downregulated genes in VS versus PR group.**

**Additional file 9. Top 10 pathways showing significant enrichment in either upregulated or downregulated genes in MS versus PR group.**

**Additional file 10. Top 10 pathways showing significant enrichment in either upregulated or downregulated genes in MS versus VS group.**

**Additional file 11. Lists of genes for thymocyte markers and thymocyte module gene sets from Gu et al.’s study [**45**].**

**Additional file 12. DEG results for thymocyte markers related to Figures **4, 5, 6, 7**.**

**Additional file 13. Co-regulation profiles of the top 3 gene sets for thymocyte modules associated with variability in fetal thymic transcriptome.**

**Additional file 14. GESECA results for thymocyte module gene sets segregated with thymocyte subsets.**

**Additional file 15. Thymocyte module gene sets significantly enriched in susceptible (VS, MS) versus resistant (CR, PR) group or between susceptible groups (MS versus VS).**

**Additional file 16. cis-ieQTL mapping results for DEGs. **First sheet: cis-ieQTLs for DEGs identified in all five contrasts. Second sheet: cis-ieQTLs for DEGs identified in four contrasts. Third sheet: cis-ieQTLs for DEGs identified in comparisons of MS vs. CR, PR, or VS. Fourth sheet: cis-ieQTLs for DEGs identified in comparisons of MS vs. CR or PR. Fifth sheet: cis-ieQTLs for DEGs identified in comparisons of VS vs. CR or PR. Sixth sheet: cis-ieQTLs for DEGs identified in only one contrast. Seventh sheet: cis-ieQTLs for DEGs identified in any remaining combinations of contrasts.

**Additional file 17. ieGenes (*N = *27) annotated with REACTOME, KEGG, PID pathways (*N = *83) significantly associated with variability in fetal thymic transcriptome.**

## Data Availability

Data used in this study are available from the corresponding author upon reasonable request.

## References

[1] Ison EK, Hopf-Jannasch AS, Harding JCS, Alex Pasternak J (2022) Effects of porcine reproductive and respiratory syndrome virus (PRRSV) on thyroid hormone metabolism in the late gestation fetus. Vet Res 53:74 36175938 10.1186/s13567-022-01092-3 PMC9524047

[2] Malgarin CM, Moser F, Pasternak JA, Hamonic G, Detmer SE, MacPhee DJ, Harding JCS (2021) Fetal hypoxia and apoptosis following maternal porcine reproductive and respiratory syndrome virus (PRRSV) infection. BMC Vet Res 17:182 33933084 10.1186/s12917-021-02883-0 PMC8088663

[3] Mulligan MK, Kleiman JE, Caldemeyer AC, Harding JCS, Pasternak JA (2022) Porcine reproductive and respiratory virus 2 infection of the fetus results in multi-organ cell cycle suppression. Vet Res 53:13 35189966 10.1186/s13567-022-01030-3 PMC8860275

[4] Pasternak JA, MacPhee DJ, Harding JCS (2020) Fetal cytokine response to porcine reproductive and respiratory syndrome virus-2 infection. Cytokine 126:154883 31629108 10.1016/j.cyto.2019.154883

[5] Pasternak JA, MacPhee DJ, Harding JCS (2020) Maternal and fetal thyroid dysfunction following porcine reproductive and respiratory syndrome virus2 infection. Vet Res 51:47 32228691 10.1186/s13567-020-00772-2 PMC7106657

[6] Van Goor A, Pasternak A, Walker K, Hong L, Malgarin C, MacPhee DJ, Harding JCS, Lunney JK (2020) Differential responses in placenta and fetal thymus at 12 days post infection elucidate mechanisms of viral level and fetal compromise following PRRSV2 infection. BMC Genomics 21:763 33148169 10.1186/s12864-020-07154-0 PMC7640517

[7] Wilkinson JM, Bao H, Ladinig A, Hong L, Stothard P, Lunney JK, Plastow GS, Harding JCS (2016) Genome-wide analysis of the transcriptional response to porcine reproductive and respiratory syndrome virus infection at the maternal/fetal interface and in the fetus. BMC Genomics 17:383 27207143 10.1186/s12864-016-2720-4 PMC4875603

[8] Malgarin CM, Macphee DJ, Harding J (2020) Fetal metabolomic alterations following porcine reproductive and respiratory syndrome virus infection. Front Mol Biosci 7:559688 33363202 10.3389/fmolb.2020.559688 PMC7759636

[9] Ashby KM, Hogquist KA (2024) A guide to thymic selection of T cells. Nat Rev Immunol 24:103–117 37464188 10.1038/s41577-023-00911-8

[10] Miller JFAP (2020) The function of the thymus and its impact on modern medicine. Science 369:eaba2429 32732394 10.1126/science.aba2429

[11] Thapa P, Farber DL (2019) The role of the thymus in the immune response. Thorac Surg Clin 29:123–131 30927993 10.1016/j.thorsurg.2018.12.001 PMC6446584

[12] Rowland RRR (2010) The interaction between PRRSV and the late gestation pig fetus. Virus Res 154:114–122 20832434 10.1016/j.virusres.2010.09.001 PMC7172144

[13] Ruedas-Torres I, Gómez-Laguna J, Sánchez-Carvajal JM, Larenas-Muñoz F, Barranco I, Pallarés FJ, Carrasco L, Rodríguez-Gómez IM (2021) Activation of T-bet, FOXP3, and EOMES in target organs from piglets infected with the virulent PRRSV-1 lena strain. Front Immunol 12:773146 34956200 10.3389/fimmu.2021.773146 PMC8697429

[14] Wang G, Yu Y, Cai X, Zhou E-M, Zimmerman JJ (2020) Effects of PRRSV infection on the porcine thymus. Trends Microbiol 28:212–223 31744664 10.1016/j.tim.2019.10.009

[15] Sinkora M, Toman M, Stepanova K, Stepanova H, Leva L, Sinkorova J, Moutelikova R, Salat J, Srutkova D, Schwarzer M, Sinkora S, Skalnikova HK, Nechvatalova K, Hudcovic T, Hermanova P, Pfeiferova S, Kratochvilova M, Kavanova L, Dusankova B, Sinkora MJ (2023) The mechanism of immune dysregulation caused by porcine reproductive and respiratory syndrome virus (PRRSV). Microb Infect 25:105146 10.1016/j.micinf.2023.105146 37142116

[16] Sinkora M, Butler JE (2009) The ontogeny of the porcine immune system. Dev Comp Immunol 33:273–283 18762210 10.1016/j.dci.2008.07.011 PMC7103207

[17] Yang T, Wilkinson J, Wang Z, Ladinig A, Harding J, Plastow G (2016) A genome-wide association study of fetal response to type 2 porcine reproductive and respiratory syndrome virus challenge. Sci Rep 6:20305 26846722 10.1038/srep20305 PMC4742883

[18] Van Goor A, Pasternak A, Walugembe M, Chehab N, Hamonic G, Dekkers JC, Harding JC, Lunney JK (2023) Genome wide association study of thyroid hormone levels following challenge with porcine reproductive and respiratory syndrome virus. Front Genet 14:1110463 36845393 10.3389/fgene.2023.1110463 PMC9947478

[19] Ko H, Sammons J, Pasternak JA, Hamonic G, Starrak G, MacPhee DJ, Detmer SE, Plastow GS, Harding JCS (2022) Phenotypic effect of a single nucleotide polymorphism on SSC7 on fetal outcomes in PRRSV-2 infected gilts. Livest Sci 255:104800

[20] Ko H, Pasternak JA, Mulligan MK, Hamonic G, Ramesh N, MacPhee DJ, Plastow GS, Harding JCS (2024) A *DIO2* missense mutation and its impact on fetal response to *PRRSV* infection. BMC Vet Res 20:255 38867209 10.1186/s12917-024-04099-4 PMC11167750

[21] Ladinig A, Wilkinson J, Ashley C, Detmer SE, Lunney JK, Plastow G, Harding JCS (2014) Variation in fetal outcome, viral load and ORF5 sequence mutations in a large scale study of phenotypic responses to late gestation exposure to type 2 porcine reproductive and respiratory syndrome virus. PLoS One 9:e96104 24756023 10.1371/journal.pone.0096104 PMC3996001

[22] Ladinig A, Ashley C, Detmer SE, Wilkinson JM, Lunney JK, Plastow G, Harding JC (2015) Maternal and fetal predictors of fetal viral load and death in third trimester, type 2 porcine reproductive and respiratory syndrome virus infected pregnant gilts. Vet Res 46:107 26407558 10.1186/s13567-015-0251-7 PMC4582889

[23] Purcell S, Chang C (2023) PLINK 1.9

[24] Chang CC, Chow CC, Tellier LCAM, Vattikuti S, Purcell SM, Lee JJ (2015) Second-generation PLINK: rising to the challenge of larger and richer datasets. Gigascience 4:7 25722852 10.1186/s13742-015-0047-8 PMC4342193

[25] Crespo-Piazuelo D, Acloque H, Gonzalez-Rodriguez O, Mongellaz M, Mercat M-J, Bink MCAM, Huisman AE, Ramayo-Caldas Y, Sanchez JP, Ballester M (2022) Identification of transcriptional regulatory variants in pig duodenum, liver, and muscle tissues. Gigascience 12:giad042 37354463 10.1093/gigascience/giad042 PMC10290502

[26] Drag MH, Kogelman LJ, Maribo H, Meinert L, Thomsen PD, Kadarmideen HN (2019) Characterization of eQTLs associated with androstenone by RNA sequencing in porcine testis. Physiol Genomics 51:488–499 31373884 10.1152/physiolgenomics.00125.2018

[27] Gonzalez-Prendes R, Quintanilla R, Amills M (2017) Investigating the genetic regulation of the expression of 63 lipid metabolism genes in the pig skeletal muscle. Anim Genet 48:606–610 28737243 10.1111/age.12586

[28] Liu Y, Liu X, Zheng Z, Ma T, Liu Y, Long H, Cheng H, Fang M, Gong J, Li X, Zhao S, Xu X (2020) Genome-wide analysis of expression QTL (eQTL) and allele-specific expression (ASE) in pig muscle identifies candidate genes for meat quality traits. Genet Sel Evol 52:59 33036552 10.1186/s12711-020-00579-x PMC7547458

[29] Ponsuksili S, Murani E, Hadlich F, Perdomo-Sabogal A, Trakooljul N, Oster M, Reyer H, Wimmers K (2022) Genetic regulation and variation of expression of miRNA and mRNA transcripts in fetal muscle tissue in the context of sex, dam and variable fetal weight. Biol Sex Differ 13:24 35550009 10.1186/s13293-022-00433-3 PMC9103043

[30] Velez-Irizarry D, Casiro S, Daza KR, Bates RO, Raney NE, Steibel JP, Ernst CW (2019) Genetic control of longissimus dorsi muscle gene expression variation and joint analysis with phenotypic quantitative trait loci in pigs. BMC Genomics 20:3 30606113 10.1186/s12864-018-5386-2 PMC6319002

[31] Ewels P, Magnusson M, Lundin S, Käller M (2016) MultiQC: summarize analysis results for multiple tools and samples in a single report. Bioinformatics 32:3047–3048 27312411 10.1093/bioinformatics/btw354 PMC5039924

[32] DePristo MA, Banks E, Poplin R, Garimella KV, Maguire JR, Hartl C, Philippakis AA, Del Angel G, Rivas MA, Hanna M (2011) A framework for variation discovery and genotyping using next-generation DNA sequencing data. Nat Genet 43:491–498 21478889 10.1038/ng.806 PMC3083463

[33] McKenna A, Hanna M, Banks E, Sivachenko A, Cibulskis K, Kernytsky A, Garimella K, Altshuler D, Gabriel S, Daly M (2010) The genome analysis toolkit: a MapReduce framework for analyzing next-generation DNA sequencing data. Genome Res 20:1297–1303 20644199 10.1101/gr.107524.110 PMC2928508

[34] Liao Y, Smyth GK, Shi W (2019) The R package Rsubread is easier, faster, cheaper and better for alignment and quantification of RNA sequencing reads. Nucleic Acids Res 47:e47 30783653 10.1093/nar/gkz114 PMC6486549

[35] Mangiola S, Molania R, Dong R, Doyle MA, Papenfuss AT (2021) Tidybulk: an R tidy framework for modular transcriptomic data analysis. Genome Biol 22:42 33482892 10.1186/s13059-020-02233-7 PMC7821481

[36] Robinson MD, Oshlack A (2010) A scaling normalization method for differential expression analysis of RNA-seq data. Genome Biol 11:R25 20196867 10.1186/gb-2010-11-3-r25 PMC2864565

[37] Gennady K, Vladimir S, Nikolay B, Boris S, Maxim NA, Alexey S (2021) Fast gene set enrichment analysis. bioRxiv:060012

[38] Zhang Y, Parmigiani G, Johnson WE (2020) ComBat-seq: batch effect adjustment for RNA-seq count data. NAR Genom Bioinform 2:lqaa078 33015620 10.1093/nargab/lqaa078 PMC7518324

[39] Liberzon A, Birger C, Thorvaldsdóttir H, Ghandi M, Mesirov JP, Tamayo P (2015) The molecular signatures database (MSigDB) hallmark gene set collection. Cell Syst 1:417–425 26771021 10.1016/j.cels.2015.12.004 PMC4707969

[40] Liberzon A, Subramanian A, Pinchback R, Thorvaldsdóttir H, Tamayo P, Mesirov JP (2011) Molecular signatures database (MSigDB) 3.0. Bioinformatics 27:1739–1740 21546393 10.1093/bioinformatics/btr260 PMC3106198

[41] Subramanian A, Tamayo P, Mootha VK, Mukherjee S, Ebert BL, Gillette MA, Paulovich A, Pomeroy SL, Golub TR, Lander ES et al (2005) Gene set enrichment analysis: a knowledge-based approach for interpreting genome-wide expression profiles. Proc Natl Acad Sci U S A 102:15545–15550 16199517 10.1073/pnas.0506580102 PMC1239896

[42] Robinson MD, McCarthy DJ, Smyth GK (2010) edgeR: a Bioconductor package for differential expression analysis of digital gene expression data. Bioinformatics 26:139–140 19910308 10.1093/bioinformatics/btp616 PMC2796818

[43] Chen Y, Lun A, Smyth G (2016) From reads to genes to pathways: differential expression analysis of RNA-seq experiments using Rsubread and the edgeR quasi-likelihood pipeline. F1000Res 5:1438 27508061 10.12688/f1000research.8987.1 PMC4934518

[44] Hart SN, Therneau TM, Zhang Y, Poland GA, Kocher J-P (2013) Calculating sample size estimates for RNA sequencing data. J Comput Biol 20:970–978 23961961 10.1089/cmb.2012.0283 PMC3842884

[45] Gu W, Madrid DMC, Joyce S, Driver JP (2022) A single-cell analysis of thymopoiesis and thymic iNKT cell development in pigs. Cell Rep 40:111050 35793622 10.1016/j.celrep.2022.111050 PMC9704770

[46] Wickham H (2016) ggplot2: elegant graphics for data analysis. Springer-Verlag, New York

[47] Zhou X, Stephens M (2012) Genome-wide efficient mixed-model analysis for association studies. Nat Genet 44:821–824 22706312 10.1038/ng.2310 PMC3386377

[48] O’Connell J, Gurdasani D, Delaneau O, Pirastu N, Ulivi S, Cocca M, Traglia M, Huang J, Huffman JE, Rudan I (2014) A general approach for haplotype phasing across the full spectrum of relatedness. PLoS Genet 10:e1004234 24743097 10.1371/journal.pgen.1004234 PMC3990520

[49] VanRaden PM (2008) Efficient methods to compute genomic predictions. J Dairy Sci 91:4414–4423 18946147 10.3168/jds.2007-0980

[50] McLaren W, Gil L, Hunt SE, Riat HS, Ritchie GRS, Thormann A, Flicek P, Cunningham F (2016) The ensembl variant effect predictor. Genome Biol 17:122 27268795 10.1186/s13059-016-0974-4 PMC4893825

[51] Aguet F, Alasoo K, Li YI, Battle A, Im HK, Montgomery SB, Lappalainen T (2023) Molecular quantitative trait loci. Nat Rev Methods Primers 3:4

[52] Teng J, Gao Y, Yin H, Bai Z, Liu S, Zeng H, PigGTEx Consortium, Bai L, Cai Z, Zhao B, Li X, Xu Z, Lin Q, Pan Z, Yang W, Yu X, Guan D, Hou Y, Keel BN, Rohrer GA, Lindholm-Perry AK, Oliver WT, Ballester M, Crespo-Piazuelo D, Quintanilla R, Canela-Xandri O, Rawlik K, Xia C, Yao Y, Zhao Q, et al (2024) A compendium of genetic regulatory effects across pig tissues. Nat Genet 56:112–123 38177344 10.1038/s41588-023-01585-7 PMC10786720

[53] Badke YM, Bates RO, Ernst CW, Schwab C, Steibel JP (2012) Estimation of linkage disequilibrium in four US pig breeds. BMC Genomics 13:24 22252454 10.1186/1471-2164-13-24 PMC3269977

[54] Ladinig A, Foxcroft G, Ashley C, Lunney JK, Plastow G, Harding JCS (2014) Birth weight, intrauterine growth retardation and fetal susceptibility to porcine reproductive and respiratory syndrome virus. PLoS ONE 9:e109541 25275491 10.1371/journal.pone.0109541 PMC4183575

[55] Boddicker N, Waide EH, Rowland RRR, Lunney JK, Garrick DJ, Reecy JM, Dekkers JCM (2012) Evidence for a major QTL associated with host response to porcine reproductive and respiratory syndrome virus challenge. J Anim Sci 90:1733–1746 22205662 10.2527/jas.2011-4464

[56] Boddicker NJ, Bjorkquist A, Rowland RR, Lunney JK, Reecy JM, Dekkers JC (2014) Genome-wide association and genomic prediction for host response to porcine reproductive and respiratory syndrome virus infection. Genet Sel Evol 46:18 24592976 10.1186/1297-9686-46-18 PMC3974599

[57] Boddicker NJ, Garrick DJ, Rowland RRR, Lunney JK, Reecy JM, Dekkers JCM (2014) Validation and further characterization of a major quantitative trait locus associated with host response to experimental infection with porcine reproductive and respiratory syndrome virus. Anim Genet 45:48–58 23914972 10.1111/age.12079

[58] Koltes JE, Fritz-Waters E, Eisley CJ, Choi I, Bao H, Kommadath A, Serão NVL, Boddicker NJ, Abrams SM, Schroyen M et al (2015) Identification of a putative quantitative trait nucleotide in guanylate binding protein 5 for host response to PRRS virus infection. BMC Genomics 16:412 26016888 10.1186/s12864-015-1635-9 PMC4446061

[59] Butler JE, Sinkora M, Wang G, Stepanova K, Li Y, Cai X (2019) Perturbation of thymocyte development underlies the PRRS pandemic: a testable hypothesis. Front Immunol 10:1077 31156633 10.3389/fimmu.2019.01077 PMC6529568

[60] Moldovan G-L, Pfander B, Jentsch S (2007) PCNA, the maestro of the replication fork. Cell 129:665–679 17512402 10.1016/j.cell.2007.05.003

[61] Witko-Sarsat V, Mocek J, Bouayad D, Tamassia N, Ribeil J-A, Candalh C, Davezac N, Reuter N, Mouthon L, Hermine O, Pederzoli-Ribeil M, Cassatella MA (2010) Proliferating cell nuclear antigen acts as a cytoplasmic platform controlling human neutrophil survival. J Exp Med 207:2631–2645 20975039 10.1084/jem.20092241 PMC2989777

[62] Park J-E, Botting RA, Domínguez Conde C, Popescu D-M, Lavaert M, Kunz DJ, Goh I, Stephenson E, Ragazzini R, Tuck E, Wilbrey-Clark A, Roberts K, Kedlian VR, Ferdinand JR, He X, Webb S, Maunder D, Vandamme N, Mahbubani KT, Polanski K, Mamanova L, Bolt L, Crossland D, de Rita F, Fuller A, Filby A, Reynolds G, Dixon D, Saeb-Parsy K, Lisgo S, Henderson D, et al. (2020) A cell atlas of human thymic development defines T cell repertoire formation. Science 367:eaay3224 32079746 10.1126/science.aay3224 PMC7611066

[63] Schäfer A, Hühr J, Schwaiger T, Dorhoi A, Mettenleiter TC, Blome S, Schröder C, Blohm U (2019) Porcine invariant natural killer T cells: functional profiling and dynamics in steady state and viral infections. Front Immunol 10:1380 31316500 10.3389/fimmu.2019.01380 PMC6611438

[64] Zhao GN, Jiang DS, Li H (2015) Interferon regulatory factors: at the crossroads of immunity, metabolism, and disease. Biochim Biophys Acta Mol Basis Dis 1852:365–378 10.1016/j.bbadis.2014.04.030 24807060

[65] Schneider WM, Chevillotte MD, Rice CM (2014) Interferon-stimulated genes: a complex web of host defenses. Annu Rev Immunol 32:513–545 24555472 10.1146/annurev-immunol-032713-120231 PMC4313732

[66] Caramalho I, Nunes-Cabaço H, Foxall RB, Sousa AE (2015) Regulatory t-cell development in the human thymus. Front Immunol 6:395 26284077 10.3389/fimmu.2015.00395 PMC4522873

[67] Lins MP (2022) Thymic extracellular matrix in the thymopoiesis: just a supporting? BioTech (Basel) 11:27 35892932 10.3390/biotech11030027 PMC9326736

[68] Herbert KM, Nag A (2016) A tale of two RNAs during viral infection: how viruses antagonize mRNAs and small non-coding RNAs in the host cell. Viruses 8:154 27271653 10.3390/v8060154 PMC4926174

[69] Qi D, Guan J, Wu E (2018) Virus infection-induced host mrna degradation and potential application of live cell imaging. Radiol Infect Dis 5:143–147 32289070 10.1016/j.jrid.2018.12.002 PMC7104030

[70] Fu W, Cheng Y, Zhang Y, Mo X, Li T, Liu Y, Wang P, Pan W, Chen Y, Xue Y, Ma D, Zhang Y, Han W (2015) The secreted form of transmembrane protein 98 promotes the differentiation of T helper 1 cells. J Interferon Cytokine Res 35:720–733 25946230 10.1089/jir.2014.0110 PMC4560856

[71] Lv G, Zhu H, Li C, Wang J, Zhao D, Li S, Ma L, Sun G, Li F, Zhao Y, Gao Y (2017) Inhibitionof IL-8-mediated endothelial adhesion, VSMCs proliferation and migration by siRNA-TMEM98 suggests TMEM98’s emerging role in atherosclerosis. Oncotarget 8:88043–88058 29152140 10.18632/oncotarget.21408 PMC5675692

[72] Lee GR (2023) Molecular mechanisms of T helper cell differentiation and functional specialization. Immune Netw 23:e4 36911803 10.4110/in.2023.23.e4 PMC9995992

[73] Berger A (2000) Th1 and Th2 responses: what are they? BMJ 321:424 10938051 10.1136/bmj.321.7258.424 PMC27457

[74] Ribatti D (2015) The discovery of the blood–thymus barrier. Immunol Lett 168:325–328 26522647 10.1016/j.imlet.2015.10.014

