# Supplementary material for: Exploring transcriptomic and genomic differences between susceptible and resistant fetal pigs to maternal PRRSV infection at late gestation
Source: Vet Res. 2025 Nov 3;56:208. doi: 10.1186/s13567-025-01621-w (PMC12584525; doi:10.1186/s13567-025-01621-w)
Supplement: Supplementary file 1 — Additional file 1. Top 20 hallmark gene sets significantly associated with variability in fetal thymic transcriptome. [file 13567_2025_1621_MOESM1_ESM.docx]

**Additional file 1.** Top 20 hallmark gene sets significantly associated with variability in fetal thymic transcriptome.

| Pathway | % Variance | BH-adjusted P | Log_2_err | Number of genes |
| --- | --- | --- | --- | --- |
| Myogenesis | 1.509 | 3.89E-81 | 2.004 | 190 |
| Interferon_gamma_response | 1.022 | 8.62E-60 | 1.721 | 183 |
| Interferon_alpha_response | 0.949 | 1.07E-45 | 1.501 | 88 |
| E2F_targets | 0.383 | 1.50E-26 | 1.148 | 199 |
| Inflammatory_response | 0.321 | 1.50E-23 | 1.075 | 186 |
| Epithelial_mesenchymal_transition | 0.326 | 3.27E-23 | 1.068 | 188 |
| G2M_checkpoint | 0.286 | 6.23E-22 | 1.040 | 194 |
| TNFA_signaling_via_NFKB | 0.290 | 5.56E-21 | 1.011 | 189 |
| Complement | 0.220 | 1.06E-16 | 0.903 | 186 |
| Allograft_rejection | 0.213 | 4.30E-16 | 0.886 | 191 |
| KRAS_signaling_up | 0.163 | 3.26E-12 | 0.770 | 185 |
| Hypoxia | 0.157 | 1.59E-11 | 0.740 | 187 |
| IL6_JAK_STAT3_signaling | 0.146 | 1.83E-11 | 0.740 | 77 |
| KRAS_signaling_dn | 0.147 | 2.37E-11 | 0.740 | 143 |
| Coagulation | 0.134 | 7.16E-11 | 0.720 | 113 |
| IL2_STAT5_signaling | 0.139 | 5.05E-10 | 0.688 | 187 |
| Apical_junction | 0.130 | 4.70E-09 | 0.655 | 184 |
| Apoptosis | 0.118 | 5.40E-09 | 0.655 | 156 |
| Oxidative_phosphorylation | 0.126 | 1.20E-08 | 0.632 | 189 |
| MYC_targets_v1 | 0.116 | 4.97E-07 | 0.571 | 197 |
