# Supplementary material for: Exploring transcriptomic and genomic differences between susceptible and resistant fetal pigs to maternal PRRSV infection at late gestation
Source: Vet Res. 2025 Nov 3;56:208. doi: 10.1186/s13567-025-01621-w (PMC12584525; doi:10.1186/s13567-025-01621-w)
Supplement: Supplementary file 2 — Additional file 2. Top 20 REACTOME and KEGG pathways from the curated gene sets significantly associated with variability in fetal thymic transcriptome. [file 13567_2025_1621_MOESM2_ESM.docx]

**Additional file 2.** **Top 20 REACTOME and KEGG pathways from the curated gene sets significantly associated with variability in fetal thymic transcriptome.**

| Subcategory | Pathway | % Variance | BH-adjusted P | Log_2_err | Number of genes |
| --- | --- | --- | --- | --- | --- |
| REACTOME | Muscle_contraction | 0.937 | 1.11E-52 | 1.637 | 162 |
| REACTOME | Striated_muscle_contraction | 1.351 | 8.97E-40 | 1.425 | 35 |
| REACTOME | Neutrophil_degranulation | 0.417 | 2.78E-29 | 1.223 | 433 |
| REACTOME | Interferon_alpha_beta_signaling | 0.616 | 7.93E-27 | 1.174 | 51 |
| REACTOME | Interferon_signaling | 0.400 | 3.00E-26 | 1.155 | 164 |
| REACTOME | Extracellular_matrix_organization | 0.364 | 1.13E-25 | 1.142 | 275 |
| REACTOME | GPCR_ligand_binding | 0.329 | 4.78E-23 | 1.082 | 266 |
| KEGG | Cytokine_cytokine_receptor_interaction | 0.321 | 9.68E-23 | 1.075 | 193 |
| REACTOME | Signaling_by_GPCR | 0.328 | 2.98E-21 | 1.040 | 480 |
| REACTOME | Class_a_1_rhodopsin_like_receptors | 0.275 | 5.46E-20 | 1.011 | 193 |
| REACTOME | Cell_cycle_mitotic | 0.323 | 1.59E-19 | 0.996 | 484 |
| KEGG | Hypertrophic_cardiomyopathy_hcm | 0.289 | 2.17E-18 | 0.966 | 73 |
| KEGG | Cardiac_muscle_contraction | 0.312 | 8.30E-18 | 0.951 | 61 |
| KEGG | Dilated_cardiomyopathy | 0.267 | 3.34E-16 | 0.911 | 76 |
| REACTOME | Mitotic_prometaphase | 0.209 | 5.92E-15 | 0.869 | 185 |
| REACTOME | Cell_cycle_checkpoints | 0.213 | 9.57E-15 | 0.861 | 252 |
| REACTOME | Cardiac_conduction | 0.200 | 6.80E-14 | 0.834 | 98 |
| REACTOME | Interferon_gamma_signaling | 0.204 | 1.59E-13 | 0.825 | 71 |
| REACTOME | Ion_homeostasis | 0.218 | 1.61E-13 | 0.825 | 47 |
| REACTOME | Peptide_ligand_binding_receptors | 0.189 | 2.17E-13 | 0.825 | 111 |
