# Supplementary material for: Exploring transcriptomic and genomic differences between susceptible and resistant fetal pigs to maternal PRRSV infection at late gestation
Source: Vet Res. 2025 Nov 3;56:208. doi: 10.1186/s13567-025-01621-w (PMC12584525; doi:10.1186/s13567-025-01621-w)
Supplement: Supplementary file 4 — Additional file 4. Co-regulation profiles of genes within the top 3 hallmark gene sets significantly associated with variability in fetal thymic transcriptome. [file 13567_2025_1621_MOESM4_ESM.docx]

**Additional file 4. Co-regulation profiles of genes within the top 3 hallmark gene sets significantly associated with variability in fetal thymic transcriptome.**


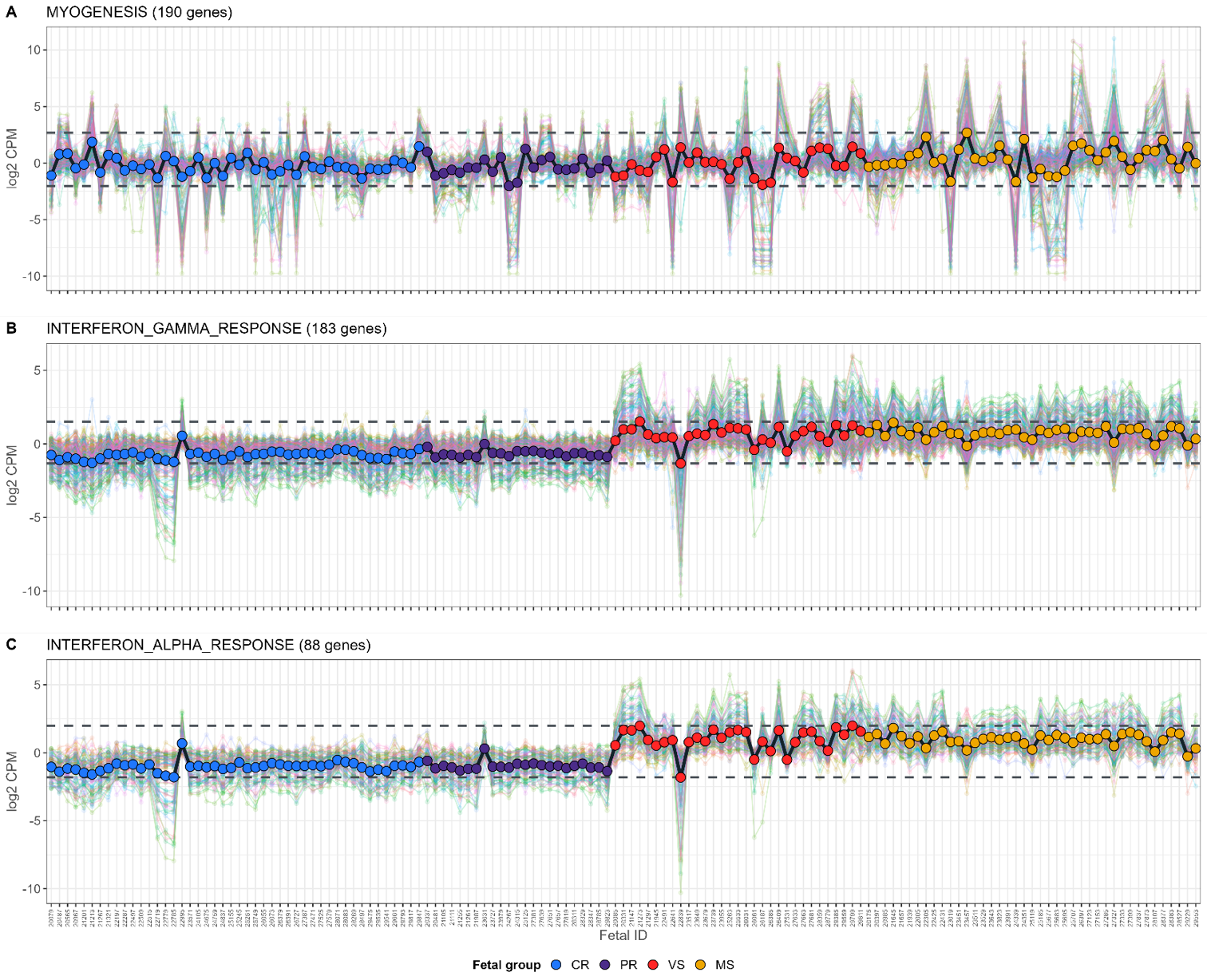


The semi-transparent lines are log_2_ CPM values centered for each gene within its respective gene set across fetal thymus samples. The gene set profile (dots colored by fetal group) was calculated for each sample (Fetal ID on x axis) as an average of the centered expression values of all genes belonging to a specific gene set within that sample. Colors denote each fetal group; Complete Resistance (CR), Partial Resistance (PR), Viable Susceptible (VS), Meconium-stained Susceptible (MS).
