# Supplementary material for: Exploring transcriptomic and genomic differences between susceptible and resistant fetal pigs to maternal PRRSV infection at late gestation
Source: Vet Res. 2025 Nov 3;56:208. doi: 10.1186/s13567-025-01621-w (PMC12584525; doi:10.1186/s13567-025-01621-w)
Supplement: Supplementary file 5 — Additional file 5. Gene expression changes in each of the six pairwise comparisons between four fetal groups (CR, PR, VS, MS). [file 13567_2025_1621_MOESM5_ESM.docx]

**Additional file 5. Gene expression changes in each of the six pairwise comparisons between four fetal groups (CR, PR, VS, MS).**


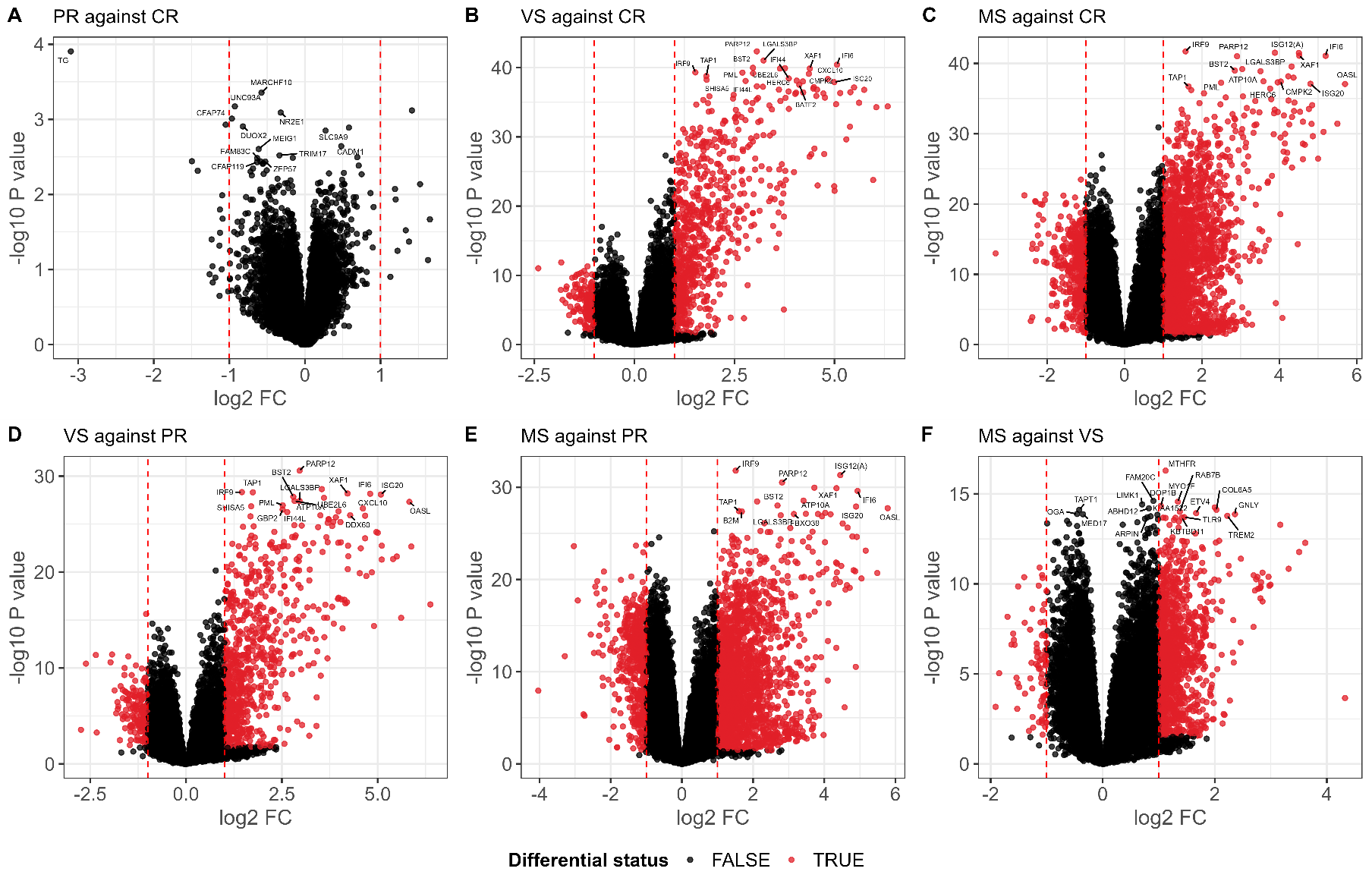


Differential status determined at False Discovery Rate (FDR) < 0.05 and log_2_ Fold Change (FC) < -1 (down-regulated) or log_2_ FC > 1 (up-regulated). Vertical red dotted lines at x = 1 and x = -1 indicate the log_2_ FC cutoffs. Top 20 annotated genes, based on the top lowest *P* values, are labelled in the plots (gene symbols); some overlapped gene symbols are removed. Each fetal group denotes Complete Resistance (CR), Partial Resistance (PR), Viable Susceptible (VS), Meconium-stained Susceptible (MS).
