# Supplementary material for: Exploring transcriptomic and genomic differences between susceptible and resistant fetal pigs to maternal PRRSV infection at late gestation
Source: Vet Res. 2025 Nov 3;56:208. doi: 10.1186/s13567-025-01621-w (PMC12584525; doi:10.1186/s13567-025-01621-w)
Supplement: Supplementary file 10 — Additional file 10. Top 10 pathways showing significant enrichment in either upregulated or downregulated genes in MS versus VS group. [file 13567_2025_1621_MOESM10_ESM.docx]

**Additional file 10. Top 10 pathways showing significant enrichment in either upregulated or downregulated genes in MS versus VS group.**


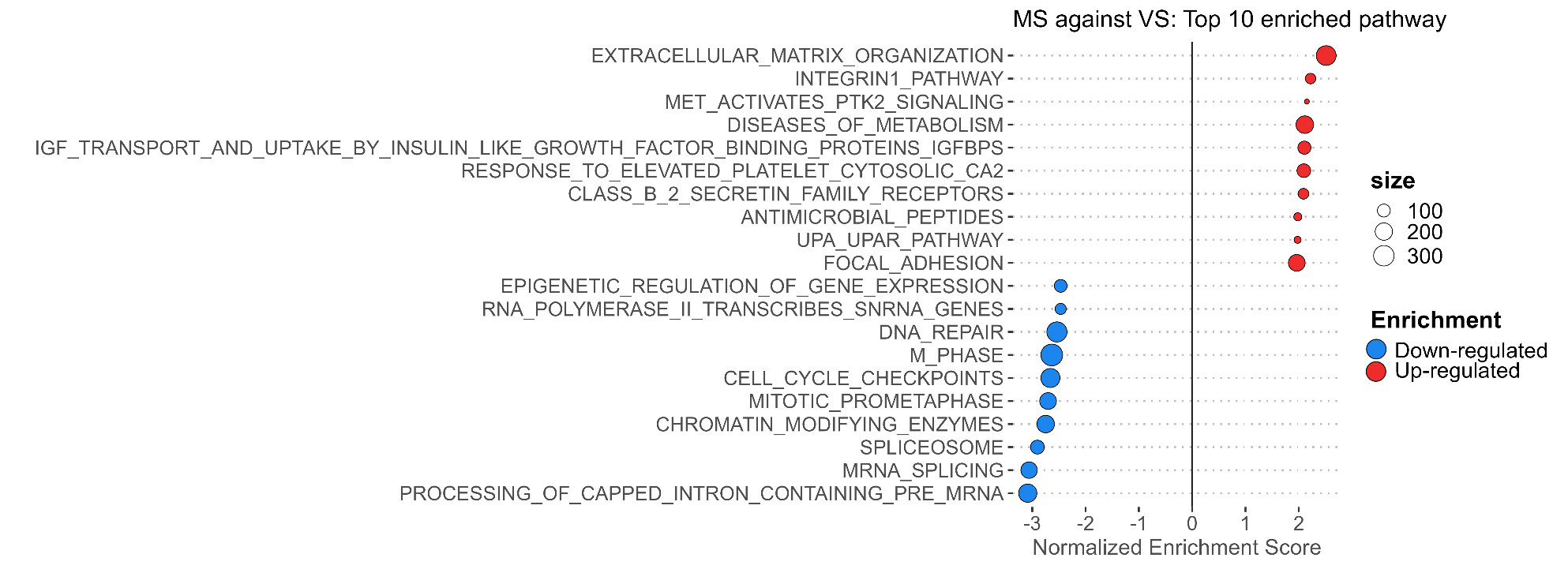


Genes were ranked by differential expression using log2 fold change and nominal *P* values (pval). Top 10 enriched pathways were selected based on normalized enrichment score adjusting for gene set size at a Benjamini-Hochberg adjusted *P* value (padj) < 0.01. In total, 47 pathways were significantly enriched among upregulated genes and 82 pathways among downregulated genes, out of 2289 tested pathways, at a padj < 0.01.
