# Supplementary material for: Exploring transcriptomic and genomic differences between susceptible and resistant fetal pigs to maternal PRRSV infection at late gestation
Source: Vet Res. 2025 Nov 3;56:208. doi: 10.1186/s13567-025-01621-w (PMC12584525; doi:10.1186/s13567-025-01621-w)
Supplement: Supplementary file 12 — Additional file 12. DEG results for thymocyte markers related to Figures 4, 5, 6, 7. [file 13567_2025_1621_MOESM12_ESM.docx]

**Additional file 12**. **DEG results for thymocyte markers related to Figures 4 to 7.**

| Gene name | Ensembl gene ID | Contrast^*^ | Log_2_ FC | FDR |
| --- | --- | --- | --- | --- |
| Double negative (DN)/double positive (DP) thymocytes | | | | |
| PCNA | ENSSSCG00000056538 | MS-CR | -1.058 | 2.12E-13 |
| PCNA | ENSSSCG00000056538 | MS-PR | -1.273 | 8.76E-14 |
| PTCRA | ENSSSCG00000001650 | MS-CR | -1.364 | 8.15E-10 |
| PTCRA | ENSSSCG00000001650 | MS-PR | -1.428 | 1.83E-08 |
| RAG1 | ENSSSCG00000026145 | MS-CR | -1.127 | 2.02E-07 |
| RAG1 | ENSSSCG00000026145 | MS-PR | -1.178 | 3.13E-06 |
| RAG1 | ENSSSCG00000026145 | MS-VS | -1.019 | 2.12E-05 |
| RAG2 | ENSSSCG00000034285 | MS-CR | -1.005 | 1.02E-06 |
| RAG2 | ENSSSCG00000034285 | MS-PR | -1.088 | 6.35E-06 |
| γδ T cells | | | | |
| BLK | ENSSSCG00000027568 | MS-CR | -1.189 | 4.75E-13 |
| BLK | ENSSSCG00000027568 | MS-PR | -1.161 | 2.60E-10 |
| CD163L1 | ENSSSCG00000034914 | MS-CR | -2.082 | 1.32E-11 |
| CD163L1 | ENSSSCG00000034914 | MS-PR | -2.339 | 1.18E-11 |
| CD163L1 | ENSSSCG00000034914 | MS-VS | -1.598 | 8.53E-07 |
| ETV5 | ENSSSCG00000023229 | VS-CR | -1.007 | 1.25E-06 |
| ETV5 | ENSSSCG00000023229 | MS-CR | -2.039 | 2.15E-20 |
| ETV5 | ENSSSCG00000023229 | VS-PR | -1.160 | 3.87E-06 |
| ETV5 | ENSSSCG00000023229 | MS-PR | -2.191 | 2.65E-19 |
| ETV5 | ENSSSCG00000023229 | MS-VS | -1.031 | 1.06E-06 |
| FHL2 | ENSSSCG00000008147 | MS-CR | -1.033 | 2.11E-08 |
| FHL2 | ENSSSCG00000008147 | MS-PR | -1.088 | 1.18E-07 |
| GATA3 | ENSSSCG00000011125 | MS-CR | -1.092 | 2.75E-14 |
| GATA3 | ENSSSCG00000011125 | MS-PR | -1.098 | 1.35E-11 |
| SOX13 | ENSSSCG00000015277 | MS-CR | -1.577 | 3.22E-20 |
| SOX13 | ENSSSCG00000015277 | MS-PR | -1.570 | 9.80E-17 |
| WC1.1 | ENSSSCG00000031085 | VS-CR | -1.091 | 6.99E-05 |
| WC1.1 | ENSSSCG00000031085 | MS-CR | -2.248 | 2.51E-16 |
| WC1.1 | ENSSSCG00000031085 | VS-PR | -1.127 | 0.000849 |
| WC1.1 | ENSSSCG00000031085 | MS-PR | -2.284 | 2.35E-14 |
| WC1.1 | ENSSSCG00000031085 | MS-VS | -1.157 | 2.43E-05 |
| Unconventional CD8+ cells | | | | |
| CCL5 | ENSSSCG00000017705 | VS-CR | 1.438 | 2.28E-10 |
| CCL5 | ENSSSCG00000017705 | MS-CR | 3.091 | 1.07E-30 |
| CCL5 | ENSSSCG00000017705 | VS-PR | 1.432 | 2.24E-07 |
| CCL5 | ENSSSCG00000017705 | MS-PR | 3.085 | 3.02E-22 |
| CCL5 | ENSSSCG00000017705 | MS-VS | 1.653 | 6.30E-11 |
| FCGRA3 | ENSSSCG00000036618 | VS-CR | 1.533 | 8.49E-10 |
| FCGRA3 | ENSSSCG00000036618 | MS-CR | 2.577 | 1.51E-22 |
| FCGRA3 | ENSSSCG00000036618 | VS-PR | 1.256 | 3.24E-05 |
| FCGRA3 | ENSSSCG00000036618 | MS-PR | 2.299 | 3.44E-14 |
| FCGRA3 | ENSSSCG00000036618 | MS-VS | 1.044 | 2.78E-05 |
| IRF7 | ENSSSCG00000012853 | VS-CR | 4.761 | 1.00E-33 |
| IRF7 | ENSSSCG00000012853 | MS-CR | 4.379 | 2.96E-31 |
| IRF7 | ENSSSCG00000012853 | VS-PR | 4.232 | 5.37E-20 |
| IRF7 | ENSSSCG00000012853 | MS-PR | 3.850 | 5.19E-19 |
| ISG15 | ENSSSCG00000040575 | VS-CR | 6.045 | 1.76E-32 |
| ISG15 | ENSSSCG00000040575 | MS-CR | 5.141 | 1.33E-28 |
| ISG15 | ENSSSCG00000040575 | VS-PR | 5.498 | 5.68E-20 |
| ISG15 | ENSSSCG00000040575 | MS-PR | 4.594 | 1.35E-17 |
| KLRB1 | ENSSSCG00000039708 | MS-CR | 1.326 | 1.15E-07 |
| KLRB1 | ENSSSCG00000039708 | MS-PR | 1.020 | 0.000674 |
| KLRK1 | ENSSSCG00000040465 | MS-CR | 2.000 | 1.02E-20 |
| KLRK1 | ENSSSCG00000040465 | MS-PR | 1.980 | 8.60E-15 |
| KLRK1 | ENSSSCG00000040465 | MS-VS | 1.289 | 6.31E-09 |
| MX1 | ENSSSCG00000012077 | VS-CR | 4.564 | 1.03E-34 |
| MX1 | ENSSSCG00000012077 | MS-CR | 3.958 | 3.73E-31 |
| MX1 | ENSSSCG00000012077 | VS-PR | 4.039 | 2.94E-21 |
| MX1 | ENSSSCG00000012077 | MS-PR | 3.432 | 8.85E-19 |
| NKG7 | ENSSSCG00000003231 | VS-CR | 1.224 | 7.69E-15 |
| NKG7 | ENSSSCG00000003231 | MS-CR | 1.699 | 1.54E-24 |
| NKG7 | ENSSSCG00000003231 | VS-PR | 1.213 | 3.39E-10 |
| NKG7 | ENSSSCG00000003231 | MS-PR | 1.688 | 9.44E-18 |
| STAT1 | ENSSSCG00000016057 | VS-CR | 2.534 | 4.01E-32 |
| STAT1 | ENSSSCG00000016057 | MS-CR | 2.109 | 4.16E-27 |
| STAT1 | ENSSSCG00000016057 | VS-PR | 2.330 | 8.17E-21 |
| STAT1 | ENSSSCG00000016057 | MS-PR | 1.905 | 1.70E-17 |
| ZBTB16 | ENSSSCG00000030095 | MS-CR | 1.909 | 2.48E-10 |
| ZBTB16 | ENSSSCG00000030095 | VS-PR | 1.036 | 0.009396 |
| ZBTB16 | ENSSSCG00000030095 | MS-PR | 2.010 | 2.83E-08 |
| Treg cells | | | | |
| CTLA4 | ENSSSCG00000016122 | VS-CR | 1.090 | 6.28E-09 |
| CTLA4 | ENSSSCG00000016122 | MS-CR | 1.087 | 3.85E-10 |
| CTLA4 | ENSSSCG00000016122 | VS-PR | 1.375 | 9.51E-09 |
| CTLA4 | ENSSSCG00000016122 | MS-PR | 1.372 | 3.76E-10 |
| S100A4 | ENSSSCG00000006578 | MS-CR | 1.100 | 1.53E-08 |
| S100A4 | ENSSSCG00000006578 | MS-PR | 1.188 | 4.34E-07 |
| TNFRSF18 | ENSSSCG00000038351 | MS-PR | 1.142 | 6.90E-08 |

^*^pairwise comparisons among the fetal groups; Complete Resistance (CR), Partial Resistance (PR), Viable Susceptible (VS), Meconium-stained Susceptible (MS). CR or PR served as the reference group when compared with VS or MS, and MS–VS denotes the comparison of MS versus VS (reference group).
