# Supplementary material for: Exploring transcriptomic and genomic differences between susceptible and resistant fetal pigs to maternal PRRSV infection at late gestation
Source: Vet Res. 2025 Nov 3;56:208. doi: 10.1186/s13567-025-01621-w (PMC12584525; doi:10.1186/s13567-025-01621-w)
Supplement: Supplementary file 13 — Additional file 13. Co-regulation profiles of the top 3 gene sets for thymocyte modules associated with variability in fetal thymic transcriptome. [file 13567_2025_1621_MOESM13_ESM.docx]

**Additional file 13. Co-regulation profiles of the top 3 gene sets for thymocyte modules associated with variability in fetal thymic transcriptome.**


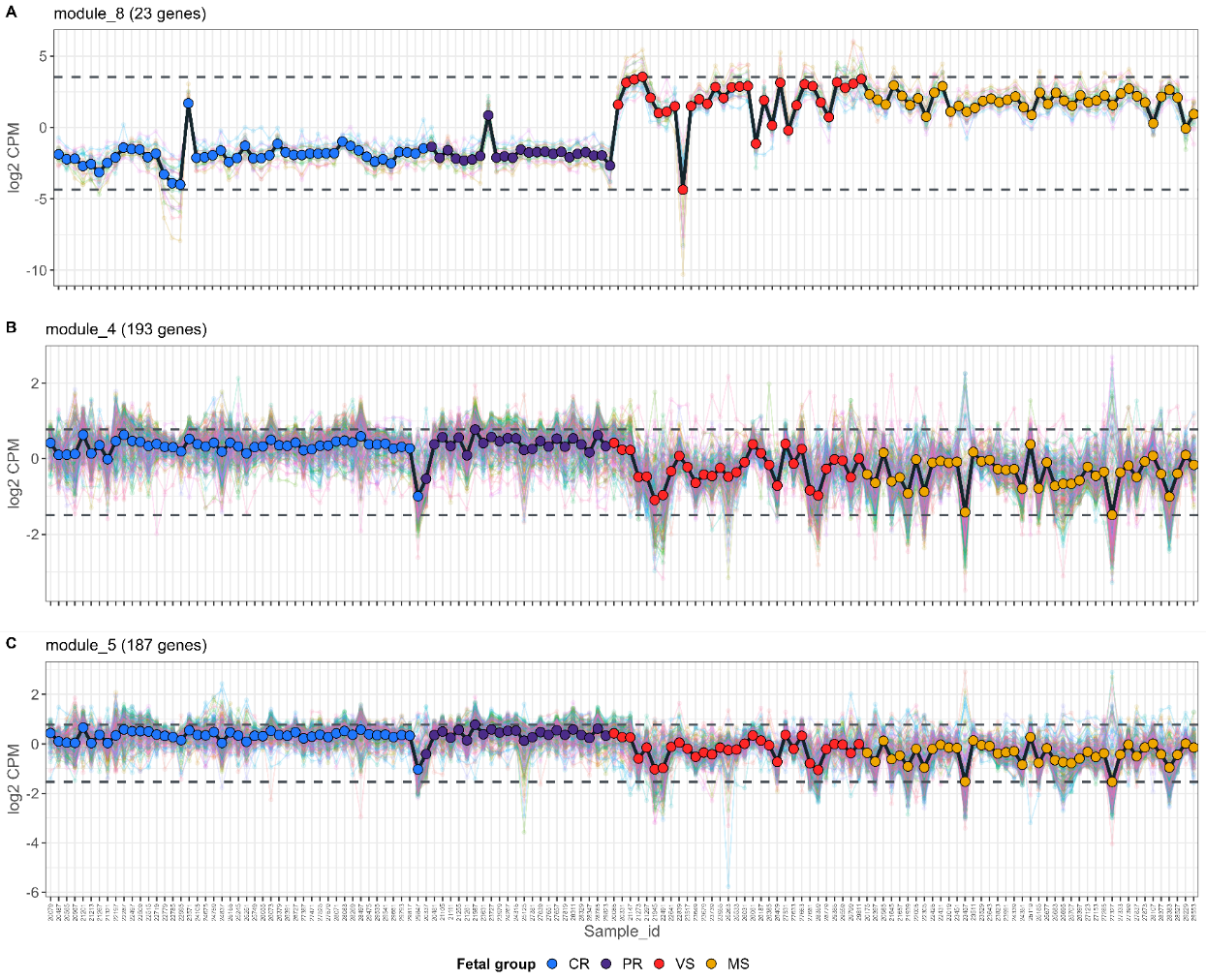


Module_8 encompasses interferon-stimulated gene (ISG)-CD8 T cells, while module_4 and _5 represent double positive (DP) thymocytes in the cell cycling stage. The semi-transparent lines are log2 CPM values centered for each gene within its respective module across fetal thymus samples. The gene set profile (dots colored by fetal group) was calculated for each sample (Sample id on x axis) as an average of the centered expression values of all genes belonging to a specific thymocyte module within that sample. Colors denote each fetal group; Complete Resistance (CR), Partial Resistance (PR), Viable Susceptible (VS), Meconium-stained Susceptible (MS).
