# Supplementary material for: Exploring transcriptomic and genomic differences between susceptible and resistant fetal pigs to maternal PRRSV infection at late gestation
Source: Vet Res. 2025 Nov 3;56:208. doi: 10.1186/s13567-025-01621-w (PMC12584525; doi:10.1186/s13567-025-01621-w)
Supplement: Supplementary file 14 — Additional file 14. GESECA results for thymocyte module gene sets segregated with thymocyte subsets. [file 13567_2025_1621_MOESM14_ESM.docx]

**Additional file 14. GESECA results for thymocyte module gene sets segregated with thymocyte subsets.**

| module | Major thymocytes  segregated | % Variance | BH-Adjusted P | Log_2_err | Number of genes |
| --- | --- | --- | --- | --- | --- |
| 8 | ISG-CD8 T cells | 0.894 | 7.16E-28 | 1.18 | 23 |
| 4 | DP(C) cells^*^ | 0.376 | 1.77E-26 | 1.142 | 193 |
| 5 | DP(C) cells | 0.371 | 7.54E-26 | 1.129 | 187 |
| 10 | CD8αα cells | 0.186 | 6.93E-14 | 0.825 | 103 |
| 7 | CD4SP, CD8SP, γδ T cells | 0.169 | 3.63E-13 | 0.798 | 132 |
| 11 | cytotoxic CD8 T cells | 0.186 | 3.02E-12 | 0.77 | 45 |
| 14 | NA^**^ | 0.195 | 4.16E-11 | 0.73 | 24 |
| 3 | DP(C) cells | 0.140 | 1.94E-10 | 0.699 | 52 |
| 9 | Treg | 0.113 | 2.84E-09 | 0.655 | 89 |
| 6 | DP(Q) cells^*^ | 0.070 | 1.05E-05 | 0.502 | 49 |
| 12 | γδ T cells | 0.068 | 3.79E-05 | 0.471 | 99 |
| 1 | DN cells | 0.060 | 0.00028 | 0.422 | 97 |
| 13 | CD2- γδ T cells | 0.029 | 0.00311 | 0.365 | 26 |
| 2 | DP(C) cells, CD2+ γδ T cells | 0.068 | 0.03985 | 0.272 | 191 |
| 15 | NA | 0.018 | 0.0618 | 0.185 | 22 |
| 16 | NA | 0.012 | 0.38761 | 0.057 | 24 |

^*^Double positive (DP) thymocytes at rapid cell cycling (C) or quiescent (Q) stage.

^**^NA, modules not segregated with specific thymocyte subsets.
