# Supplementary material for: Exploring transcriptomic and genomic differences between susceptible and resistant fetal pigs to maternal PRRSV infection at late gestation
Source: Vet Res. 2025 Nov 3;56:208. doi: 10.1186/s13567-025-01621-w (PMC12584525; doi:10.1186/s13567-025-01621-w)
Supplement: Supplementary file 15 — Additional file 15. Thymocyte module gene sets significantly enriched in susceptible (VS, MS) versus resistant (CR, PR) group or between susceptible groups (MS versus VS). [file 13567_2025_1621_MOESM15_ESM.docx]

**Additional file 15.** Thymocyte module gene sets significantly enriched in susceptible (VS, MS) versus resistant (CR, PR) group or between susceptible groups (MS versus VS).


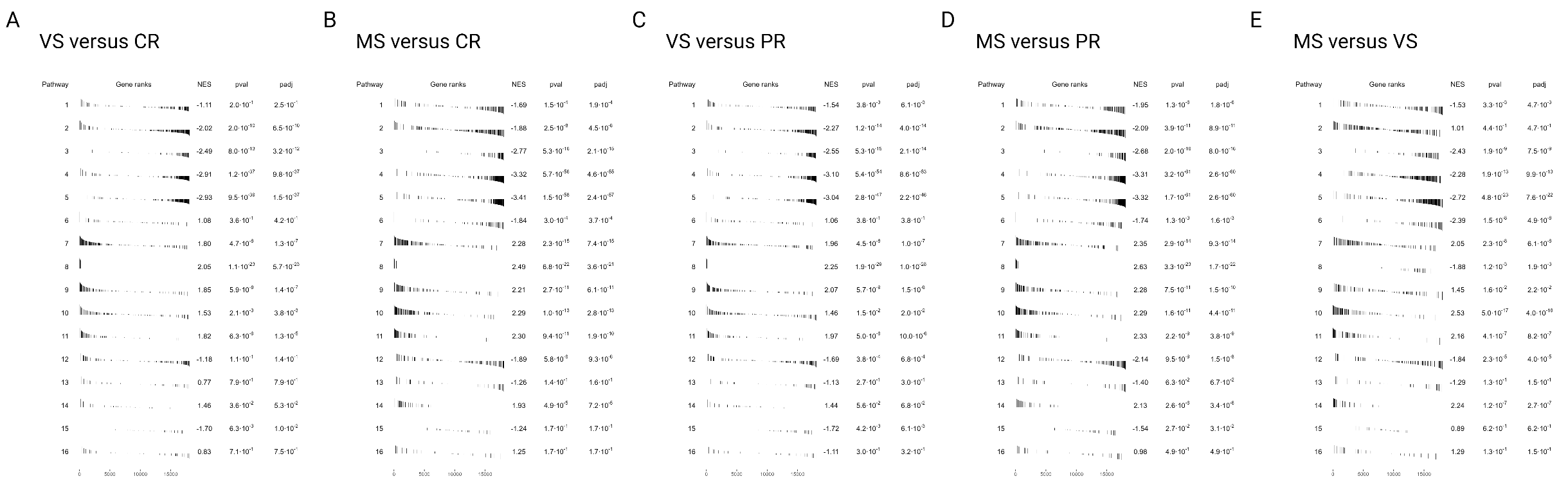


(A-E) Thymic expressed genes (N=18 100) were ranked by differential expression using log2 fold change (FC) and nominal *P* values (pval) on X axis. Genes with the highest up or downregulation based on log2 FC were ranked at the top or bottom X axis. Significantly enriched pathways were defined based on a Benjamini-Hochberg adjusted P value (padj) < 0.1. NES, normalized enrichment score adjusting for gene set size. Pathways consist of module 1 to 16, based on pseudo-temporal analysis, reflecting distinct developmental stages of specific thymocyte subsets, except module 14-16, as follows: developing thymocytes (double negative, DN (module 1) and double positive (DP) thymocytes either at cell cycling stage (module 2-5) or quiescent (module 6)), post-committed thymocytes (conventional T cells, CD4+/CD8+ T cells (module 7); unconventional CD8+ T cells, interferon-stimulated gene (ISG)-CD8 T cells (module 8), CD8αα cells (module 10), cytotoxic CD8 T cells (module11); T regulatory cells (module 9), and CD2+ (module 2,7,12)/CD2- γδ T cells (module 7,12,13).
