# Supplementary material for: Exploring transcriptomic and genomic differences between susceptible and resistant fetal pigs to maternal PRRSV infection at late gestation
Source: Vet Res. 2025 Nov 3;56:208. doi: 10.1186/s13567-025-01621-w (PMC12584525; doi:10.1186/s13567-025-01621-w)
Supplement: Supplementary file 17 — Additional file 17. ieGenes (N = 27) annotated with REACTOME, KEGG, PID pathways (N = 83) significantly associated with variability in fetal thymic transcriptome. [file 13567_2025_1621_MOESM17_ESM.docx]

**Additional file 17.** ieGenes (N=27) annotated with REACTOME, KEGG, PID pathways (N=83) significantly associated with variability in fetal thymic transcriptome.

| Subcategory | Pathway | % Variance | P value | Adjusted P | Log2err | Number of genes | ieGene |
| --- | --- | --- | --- | --- | --- | --- | --- |
| REACTOME | Muscle_contraction | 0.937 | 2.84E-55 | 1.11E-52 | 1.637 | 162 | *SCN2B* |
| REACTOME | Neutrophil_degranulation | 0.417 | 3.97E-31 | 2.78E-29 | 1.223 | 433 | *HK3, MNDA, MPO, SERPINB10* |
| REACTOME | Interferon_alpha_beta_signaling | 0.616 | 1.46E-28 | 7.93E-27 | 1.174 | 51 | *GBP2* |
| REACTOME | Interferon_signaling | 0.400 | 6.13E-28 | 3.00E-26 | 1.155 | 164 | *GBP2, LOC102161784* |
| REACTOME | Extracellular_matrix_organization | 0.364 | 2.60E-27 | 1.13E-25 | 1.142 | 275 | *COL15A1, ICAM4* |
| REACTOME | Gpcr_ligand_binding | 0.329 | 1.46E-24 | 4.78E-23 | 1.082 | 266 | *CCR10, CXCL10, CXCL11, CXCL9* |
| KEGG | Cytokine_cytokine_receptor_interaction | 0.321 | 3.12E-24 | 9.68E-23 | 1.075 | 193 | *CCR10, CXCL10, CXCL11, CXCL9* |
| REACTOME | Signaling_by_gpcr | 0.328 | 1.10E-22 | 2.98E-21 | 1.040 | 480 | *CCR10, CXCL10, CXCL11, CXCL9* |
| REACTOME | Class_a_1_rhodopsin_like_receptors | 0.275 | 2.44E-21 | 5.46E-20 | 1.011 | 193 | *CCR10, CXCL10, CXCL11, CXCL9* |
| REACTOME | Cell_cycle_mitotic | 0.323 | 7.55E-21 | 1.59E-19 | 0.996 | 484 | *DYNC1I1* |
| REACTOME | Mitotic_prometaphase | 0.209 | 4.66E-16 | 5.92E-15 | 0.869 | 185 | *DYNC1I1* |
| REACTOME | Cell_cycle_checkpoints | 0.213 | 7.97E-16 | 9.57E-15 | 0.861 | 252 | *DYNC1I1* |
| REACTOME | Cardiac_conduction | 0.200 | 6.36E-15 | 6.80E-14 | 0.834 | 98 | *SCN2B* |
| REACTOME | Interferon_gamma_signaling | 0.204 | 1.52E-14 | 1.59E-13 | 0.825 | 71 | *GBP2, LOC102161784* |
| REACTOME | Peptide_ligand_binding_receptors | 0.189 | 2.18E-14 | 2.17E-13 | 0.825 | 111 | *CCR10, CXCL10, CXCL11, CXCL9* |
| REACTOME | Resolution_of_sister_chromatid_cohesion | 0.174 | 6.25E-14 | 5.69E-13 | 0.807 | 113 | *DYNC1I1* |
| REACTOME | M_phase | 0.212 | 1.82E-13 | 1.56E-12 | 0.798 | 346 | *DYNC1I1* |
| REACTOME | Immunoregulatory_interactions_between_a_lymphoid_and_a_non_lymphoid_cell | 0.173 | 6.89E-13 | 5.35E-12 | 0.779 | 96 | *CD300LG, ICAM4, LOC100523789* |
| REACTOME | Chemokine_receptors_bind_chemokines | 0.182 | 1.50E-12 | 1.13E-11 | 0.770 | 43 | *CCR10, CXCL10, CXCL11, CXCL9* |
| KEGG | Calcium_signaling_pathway | 0.147 | 9.01E-12 | 6.08E-11 | 0.740 | 143 | *SPHK1* |
| REACTOME | Degradation_of_the_extracellular_matrix | 0.150 | 1.27E-11 | 8.41E-11 | 0.740 | 127 | *COL15A1* |
| REACTOME | Collagen_formation | 0.145 | 2.44E-11 | 1.58E-10 | 0.730 | 87 | *COL15A1* |
| REACTOME | Interleukin_10_signaling | 0.160 | 3.16E-11 | 2.02E-10 | 0.720 | 38 | *CXCL10* |
| REACTOME | Mitotic_spindle_checkpoint | 0.140 | 4.21E-11 | 2.65E-10 | 0.720 | 106 | *DYNC1I1* |
| REACTOME | G_alpha_i_signalling_events | 0.152 | 1.14E-10 | 6.88E-10 | 0.699 | 209 | *CCR10, CXCL10, CXCL11, CXCL9* |
| REACTOME | Separation_of_sister_chromatids | 0.134 | 1.70E-10 | 9.92E-10 | 0.699 | 176 | *DYNC1I1* |
| REACTOME | Mitotic_metaphase_and_anaphase | 0.151 | 2.61E-10 | 1.51E-09 | 0.688 | 221 | *DYNC1I1* |
| REACTOME | Complement_cascade | 0.134 | 3.94E-10 | 2.23E-09 | 0.688 | 43 | *C1R* |
| REACTOME | Signaling_by_interleukins | 0.197 | 4.65E-10 | 2.61E-09 | 0.678 | 394 | *CXCL10* |
| KEGG | Toll_like_receptor_signaling_pathway | 0.113 | 2.73E-09 | 1.41E-08 | 0.655 | 89 | *CXCL10, CXCL11, CXCL9* |
| KEGG | Cell_adhesion_molecules_cams | 0.109 | 5.96E-09 | 2.89E-08 | 0.644 | 107 | *NECTIN1* |
| REACTOME | Collagen_biosynthesis_and_modifying_enzymes | 0.115 | 7.06E-09 | 3.36E-08 | 0.632 | 65 | *COL15A1* |
| REACTOME | Assembly_of_collagen_fibrils_and_other_multimeric_structures | 0.098 | 1.35E-08 | 6.17E-08 | 0.632 | 58 | *COL15A1* |
| KEGG | Chemokine_signaling_pathway | 0.116 | 1.73E-08 | 7.64E-08 | 0.621 | 157 | *CCR10, CXCL10, CXCL11, CXCL9* |
| REACTOME | Rho_gtpases_activate_formins | 0.111 | 2.13E-08 | 9.22E-08 | 0.621 | 127 | *DYNC1I1* |
| REACTOME | Integrin_cell_surface_interactions | 0.100 | 2.48E-08 | 1.06E-07 | 0.621 | 80 | *ICAM4* |
| KEGG | Complement_and_coagulation_cascades | 0.111 | 2.52E-08 | 1.08E-07 | 0.621 | 48 | *C1R* |
| KEGG | Cytosolic_dna_sensing_pathway | 0.096 | 6.72E-08 | 2.66E-07 | 0.596 | 41 | *CXCL10* |
| REACTOME | Collagen_degradation | 0.089 | 1.82E-07 | 6.80E-07 | 0.584 | 62 | *COL15A1* |
| KEGG | Parkinsons_disease | 0.091 | 2.30E-07 | 8.43E-07 | 0.571 | 113 | *SEPTIN5* |
| REACTOME | Collagen_chain_trimerization | 0.089 | 2.68E-07 | 9.70E-07 | 0.571 | 42 | *COL15A1* |
| REACTOME | Cell_cell_communication | 0.085 | 8.57E-07 | 2.84E-06 | 0.558 | 108 | *NECTIN1* |
| KEGG | Graft_versus_host_disease | 0.086 | 1.09E-06 | 3.54E-06 | 0.544 | 25 | *LOC100523789* |
| REACTOME | Initial_triggering_of_complement | 0.093 | 1.39E-06 | 4.40E-06 | 0.544 | 21 | *C1R* |
| REACTOME | Neuronal_system | 0.136 | 1.64E-06 | 5.12E-06 | 0.544 | 290 | *KCNH3* |
| PID | Integrin3_pathway | 0.068 | 2.11E-06 | 6.42E-06 | 0.531 | 40 | *SPHK1* |
| REACTOME | Cell_junction_organization | 0.072 | 3.17E-06 | 9.34E-06 | 0.531 | 75 | *NECTIN1* |
| KEGG | Rig_i_like_receptor_signaling_pathway | 0.066 | 3.78E-06 | 1.09E-05 | 0.516 | 56 | *CXCL10, DHX58* |
| KEGG | Intestinal_immune_network_for_iga_production | 0.061 | 6.32E-06 | 1.74E-05 | 0.516 | 40 | *CCR10* |
| REACTOME | DDX58_IFIH1_mediated_induction_of_interferon_alpha_beta | 0.070 | 6.52E-06 | 1.80E-05 | 0.516 | 69 | *DHX58* |
| REACTOME | Sensory_perception | 0.089 | 8.51E-06 | 2.28E-05 | 0.502 | 154 | *GUCA1A, SCN2B* |
| KEGG | Antigen_processing_and_presentation | 0.062 | 1.85E-05 | 4.61E-05 | 0.487 | 49 | *LOC100523789* |
| PID | Avb3_integrin_pathway | 0.060 | 2.85E-05 | 6.75E-05 | 0.487 | 71 | *ADGRA2, COL15A1* |
| PID | IL23_pathway | 0.055 | 3.18E-05 | 7.45E-05 | 0.471 | 31 | *CXCL9, MPO* |
| KEGG | Natural_killer_cell_mediated_cytotoxicity | 0.065 | 4.25E-05 | 9.61E-05 | 0.471 | 91 | *LOC100523789* |
| KEGG | Systemic_lupus_erythematosus | 0.053 | 6.06E-05 | 0.000132 | 0.455 | 58 | *C1R* |
| PID | Syndecan_1_pathway | 0.054 | 7.37E-05 | 0.000157 | 0.455 | 45 | *COL15A1* |
| REACTOME | Signaling_by_receptor_tyrosine_kinases | 0.175 | 0.000108 | 0.000221 | 0.455 | 472 | *SPHK1* |
| REACTOME | Phase_0_rapid_depolarisation | 0.046 | 0.000135 | 0.000271 | 0.439 | 25 | *SCN2B* |
| REACTOME | Potassium_channels | 0.055 | 0.000156 | 0.000309 | 0.439 | 62 | *KCNH3* |
| REACTOME | Visual_phototransduction | 0.053 | 0.000201 | 0.000389 | 0.439 | 70 | *GUCA1A* |
| REACTOME | ROS_and_RNS_production_in_phagocytes | 0.041 | 0.00053 | 0.000907 | 0.404 | 31 | *MPO* |
| REACTOME | DAP12_interactions | 0.040 | 0.00063 | 0.001064 | 0.404 | 33 | *LOC100523789* |
| REACTOME | Interaction_between_L1_and_ankyrins | 0.039 | 0.000703 | 0.001169 | 0.404 | 26 | *SCN2B* |
| PID | Integrin2_pathway | 0.036 | 0.000869 | 0.001411 | 0.404 | 23 | *ICAM4* |
| REACTOME | Cell_cell_junction_organization | 0.042 | 0.000869 | 0.001411 | 0.404 | 48 | *NECTIN1* |
| KEGG | Glycolysis_gluconeogenesis | 0.039 | 0.001584 | 0.002391 | 0.385 | 48 | *HK3* |
| PID | AP1_pathway | 0.043 | 0.001625 | 0.002444 | 0.385 | 67 | *ATF3* |
| REACTOME | Response_of_EIF2AK4_GCN2_to_amino_acid_deficiency | 0.051 | 0.002429 | 0.00348 | 0.365 | 96 | *ATF3* |
| REACTOME | Post_translational_modification_synthesis_of_GPI_anchored_proteins | 0.043 | 0.002482 | 0.003541 | 0.365 | 71 | *PRND* |
| REACTOME | Sphingolipid_metabolism | 0.045 | 0.004067 | 0.005496 | 0.344 | 82 | *SPHK1* |
| REACTOME | Adherens_junctions_interactions | 0.030 | 0.006329 | 0.008122 | 0.344 | 28 | *NECTIN1* |
| REACTOME | Sensory_perception_of_taste | 0.024 | 0.007838 | 0.009757 | 0.322 | 18 | *SCN2B* |
| REACTOME | Signaling_by_VEGF | 0.047 | 0.009676 | 0.011688 | 0.322 | 99 | *SPHK1* |
| KEGG | Starch_and_sucrose_metabolism | 0.026 | 0.015731 | 0.017744 | 0.298 | 31 | *HK3* |
| REACTOME | Extra_nuclear_estrogen_signaling | 0.035 | 0.018552 | 0.020489 | 0.298 | 67 | *SPHK1* |
| KEGG | Sphingolipid_metabolism | 0.025 | 0.021156 | 0.023001 | 0.298 | 36 | *SPHK1* |
| REACTOME | DAP12_signaling | 0.022 | 0.025061 | 0.026568 | 0.298 | 27 | *LOC100523789* |
| REACTOME | L1CAM_interactions | 0.044 | 0.027882 | 0.029289 | 0.298 | 104 | *SCN2B* |
| PID | Nectin_pathway | 0.023 | 0.028967 | 0.030282 | 0.298 | 29 | *NECTIN1* |
| REACTOME | Signaling_by_retinoic_acid | 0.024 | 0.031138 | 0.03231 | 0.272 | 35 | *ALDH8A1* |
| PID | S1P_S1P1_pathway | 0.019 | 0.033316 | 0.034029 | 0.272 | 21 | *SPHK1* |
| REACTOME | Voltage_gated_potassium_channels | 0.021 | 0.034187 | 0.034845 | 0.272 | 23 | *KCNH3* |
